# Component-Based Interactive Framework for Intelligent Transportation Cyber-Physical Systems

**DOI:** 10.3390/s20010264

**Published:** 2020-01-02

**Authors:** Sangsoo Jeong, Youngmi Baek, Sang H. Son

**Affiliations:** Information and Communication Engineering, DGIST, Daegu 42988, Korea; 88jeongss@dgist.ac.kr (S.J.); son@dgist.ac.kr (S.H.S.)

**Keywords:** cyber-physical systems, framework, interaction, coexistence, behavior model, human and hardware in the loop system

## Abstract

While emerging technology for self-driving automation in vehicles progresses rapidly, the transition to an era of roads full of fully connected and automated vehicles (CAVs) may take longer than expected. Until then, it is inevitable that CAVs should coexist and interact with drivers of non-autonomous vehicles (NAVs) in urban roads. During this period of transition, it is critical to provide road safety with the mixed vehicular traffic and uncertainty caused by human drivers. To investigate the issues caused by the coexistence and interaction with humans, we propose to build a component-based and interactive intelligent transportation cyber-physical systems (ITCPS) framework. Our design of the interactive ITCPS framework aims to provide a standardized structure for users by defining core components. The framework is specified by behavior models and interfaces for the desired ITCPS components and is implemented as a form of human and hardware-in-the-loop system. We developed an intersection crossing assistance service and an automatic emergency braking service as an example of practical applications using the framework. To evaluate the framework, we tested its performance to show how effectively it operates while supporting real-time processing. The results indicate that it satisfies the timing requirements of vehicle-to-vehicle (V2V) communication and the limited processing time required for performing the functions of behavior models, even though the traffic volume reaches the road capacity. A case study using statistical analysis is conducted to assess the practical value of the developed experimental environment. The results of the case study validate the reliability among the specified variables for the experiments involving human drivers. It has shown that V2V communication support has positive effects on road safety, including intersection safety, braking events, and perception-reaction time (PRT) of the drivers. Furthermore, V2V communication support and PRT are identified as the important indicators affecting road safety at an un-signalized intersection. The proposed interactive framework is expected to contribute in constructing a comprehensive environment for the urban ITCPS and providing experimental support for the analysis of human behavior in the coexistence environment.

## 1. Introduction

One of the significant strengths of cyber-physical systems (CPS) is the ability to carry out real-time interaction in the way that all components of the CPS naturally take part in the ongoing feedback loop [1]. Cooperative intelligent transportation systems (C-ITS) is an emerging CPS that enable vehicles to interact with each other, road infrastructure, and users through wireless connectivity technology. This interaction allows all components of C-ITS to actively coordinate their actions by sharing the required information in real time. In this regard, C-ITS is referred to as intelligent transportation CPS (ITCPS). We are moving towards an era of self-driving automation based on ITCPS. There is no doubt that in the future, the roads would be full of autonomous vehicles (AVs). It is expected that, after 2030, cooperative intelligent transport infrastructure and AVs will be rapidly deployed on the public roads [2,3]. Nevertheless, some experts are skeptical about the time when such a vision is fully realized [4]. They claim that it would take much longer to fill the road with only AVs. According to a survey regarding to driver’s acceptance, many drivers still feel uncomfortable and resist a series of changes with fully AVs [5,6,7]. Besides, without nudges from government and attractive price incentives, it will take a long time for people to stop driving completely [8]. Therefore, a period of long transition will exist until only fully AVs are legitimately deployed on urban roads.

The distinctive characteristics of the transition period are coexistence and interaction. The Society of Automotive Engineers (SAE) automation levels adopted by the National Highway Traffic Safety Administration (NHTSA) defines an AV to have Level 3 (L3) of automation, which indicates conditional driving automation [2,3]. For traveling, a human driver of the L3 AV is ready to take over the control of the AV at all times with notice. Nevertheless, the majority of vehicles will be still controlled by the human drivers along with other AVs. They are human-driven vehicles with either Level 0 (no driving automation), Level 1 (driver assistance), or Level 2 (partial driving automation) of the automation. In this paper, they are referred to as non-autonomous vehicles (NAV). Such a transition period is a tricky time because of uncertainty caused by the coexistence of AVs and NAVs. Unexpected and fatal crashes in a coexistence situation might happen even though all AVs comply strictly with the transportation rules. For instance, on 1 July 2015, the accident of the Google’s self-driving car indicates that the responsibility of this accident lies in an error of judgment of the human driver [9]. On the contrary, on 14 February 2016, the Google’s self-driving car itself caused the first crash even though it had used all of the sensed data and had decided that it was safe to go that way, not considering the behavior of the bus driver that led to the accident [10]. It has shown the incompleteness of the decision process of the Google’s car.

It is clear that there is still a long way to go to prepare the new era of coexistence because of high uncertainty of the road environment with the mixed traffic, the dynamic nature of the ITCPS, a lack of the smart transport facilities, the ineffective penetration rate of the AVs, and the limitation of post-crash analysis [11]. Human factors related to driving become complicated since it is hard to predict driver’s behavior on the road. Especially, drivers’ different responses on this coexistence might also lead to a change in a traffic condition. Nevertheless, in transportation research, a human driver is considered only as part of vehicle automation [12,13,14,15,16,17,18,19,20]. With regard to human behavior, many human factors studies have been conducted within a limited scope of driver adaptation and acceptance of AVs. A virtual ITCPS environment is expected to provide an effective tool to investigate interaction between AVs and NAVs, including human factors.

The ITCPS environment is a complex system that is integrated with various technologies developed from multidisciplinary domains. The desired functions and the desired levels of the functionality are different, respectively, for each research domain, resulting in a variety of complex requirements. To deal with the complexity, the ITCPS environment should aim to provide a standardized structure for users by defining core components required for building a general ITCPS environment, including coexistence and interaction. However, the literature survey indicates that, to construct a desired ITCPS environment, different models need to be individually developed [13,19,20,21,22,23]. In each model, the elements and functions are limited to its own purpose specified in each research domain. Furthermore, to the best of our knowledge until now, the coordination among different environments required for building the ITCPS environment with coexistence and interaction has not yet been described in a top-down approach [24]. Many existing studies focus on construction of the integrated environment in terms of implementation [22,25,26,27]. To tackle the challenges and help reduce potential risks of the ITCPS, we propose a novel interactive framework for an urban ITCPS considering coexistence. The coexistence indicates that a human-driven vehicle with the automation under Level 2 (L2) exists simultaneously with an autonomous vehicle over Level 3 (L3). To design an interactive ITCPS framework, a component-based approach is used. An interactive ITCPS framework is assembled with the components representing users, vehicles, and transport infrastructures. To support drivers and real-time interactions, we build a human and hardware-in-the-loop system (H2iLS), which consists of a cyber environment, a physical environment, and an added loop for drivers’ feedback. In order to validate the effectiveness of the ITCPS framework, we present two types of experimental results. First, we evaluate its performance to determine if the designed framework meets the functional requirements. Second, a case study is conducted to show its capability to provide reliable and meaningful data for identifying and solving inherent problems. In a small-scale investigation in which 12 people take part, the statistical analysis identifies important factors that may affect the perception–reaction time and intersection safety.

The main contribution of this work is the design of the ITCPS framework considering a mixed traffic environment. In addition, the interactive ITCPS framework is distinguished from existing transportation environments as it focuses on a real-time interactive approach to synchronizing individual behaviors over vehicle-to-vehicle (V2V) communication. The effectiveness of our framework is demonstrated by a statistical analysis-based case study using the H2iLS. The results indicate that an interactive ITCPS framework is useful in providing core components for constructing diverse ITCPS environments for a wide range of transport applications.

The remainder of this paper is organized as follows. In Section 2, we discuss the problems and requirements for ITCPS and review the existing studies. We present the overall design of the interactive ITCPS framework and explain the implementation of the main components in Section 3. In Section 4, we evaluate the performance of the realized framework. Section 5 describes the practical case study to show the effectiveness of the interactive ITCPS framework. Finally, the conclusion and future work are provided in Section 6.

## 2. Problem Description and Background on Intelligent Transportation Cyber-Physical Systems

In this section, we discuss the requirements imposed on the ITCPS framework design and present our approach. We also review previous studies to highlight the difference between our framework and other work.

### 2.1. Requirements for an Interactive ITCPS Framework

An ITCPS environment is achieved by tightly combining the key components of transportation systems that typically consist of road infrastructure, road vehicles, and road users [28]. The road infrastructure of the ITCPS framework includes traffic management policies and transport facilities. A traffic management policy refers to an active strategy responding to diverse and ever-changing traffic conditions. A road user of the ITCPS is generally referred to as drivers, passengers, and pedestrians. Among them, in this paper, we consider only a human driver as a road user unless otherwise specified. Two important features in the design of an interactive ITCPS environment are coexistence and interactions with human drivers. To study issues in a coexistence situation, we consider two types of road vehicles for the generation of traffic flow: CAVs (connected and automated vehicles) and CNAVs (connected and non-automated vehicles). We discuss the design requirements from three perspectives: A transportation environment, coexistence, and real-time interaction.

First, a transportation environment. From the perspective of a road user, a well-coordinated environment enabling intelligent road-traffic management services of C-ITS is critical. This is because, in a virtual environment, the system can generate reliable results only when the driving environment is similar to that of the actual traveling for a user. In this regard, it is necessary for an interactive ITCPS framework to tackle three issues: (1) Support for intelligent road-traffic management services, (2) visualization of the driving environment for a realistic driving experience, and (3) provision of longitudinal interaction for natural vehicular movements. We briefly discuss each issue as follows:

- The interactive ITCPS framework should allow a set of intelligent road-traffic management services of C-ITS. Such services include traffic management and traffic information services in the road infrastructure. The traffic management and traffic information services contribute to maximizing the road efficiency by proactively eliminating congestion and increasing the number of vehicles passing through a given road section [29]. In order to support proactive traffic management, an autonomous intersection management (AIM) method is required for vehicular traffic control at a specific intersection without a traffic light. In addition, the traffic management supports the management of the trajectory along with the designated path. Collecting and providing traffic information can be performed by V2V communication within the ITCPS.
- Representing various characteristics of the transport facilities in the road infrastructure should be enhanced with three-dimensional (3D) visualization. Elaborate visualization enables a road user to immerse into a realistic driving environment, which should be the same as one would expect to experience in the real road.
- To construct a virtual world corresponding with the real driving world, natural vehicle movements are required. For instance, all vehicles should move naturally, change the speeds responding to the current traffic flow, and maintain the safety distance to the preceding vehicle. Before and after the lane change, the movement of tail-end vehicles should respond accordingly. All vehicles of the interactive framework should be handled independently to reflect the effect of interaction. Therefore, to maneuver vehicles with longitudinal movements, a microscopic traffic model is well suited for traffic control.

Second, the coexistence. All vehicles of the interactive ITCPS framework should have the capability to connect to any vehicle. When interesting services based on particular technologies become prevalent, it is expected that road users purchase the useful equipment as shown in the case of universalization of navigation systems [30]. Since the road users of NAVs prefer an affordable solution to receive efficient and convenient road services, it is expected that they purchase cheaper third-party wireless communications equipment to conduct interactions, rather than purchasing expensive vehicles enabling a higher level of automation. Therefore, although the automation of NAVs is at or below L2, these NAVs are expected to have the ability to communicate with other vehicles through V2X communication. In this paper, we assume that the CNAV is not equipped with any sensor.

Third, real-time interaction between CAVs and CNAVs. Connected technology is essential for the AVs in future ITCPS. During the early stage of the development of ITCPS towards full automation, exchanging information over a seamless wireless connection is highly effective to provide enhanced safety and efficiency for users. In detail, the communication aims to exchange basic safety messages (BSMs) regarding vehicle states, e.g., acceleration, heading, vehicle position, vehicle size, and the states of the safety-related systems. Therefore, V2V communication enables interaction between CNAVs as well as CNAVs and CAVs. Furthermore, it is critical to provide real-time closed-loop feedback among the user, vehicle, and road infrastructure components. The driver’s behavior and response as input can change the surrounding traffic situation, and then the changed traffic situation may have an unexpected effect on overall traffic flow. At the same time, the behavior of one driver can also affect and change the driving decisions of nearby drivers, and the consequences affect the driver again. The behavior is related to certain maneuvers such as pushing down an acceleration pedal or a brake pedal, turning a steering wheel, or shifting gears. As output of the behavior, this change in the situation affects the driver’s next decision and reaction. The problem is that it is difficult to clearly model and identify how behaviors of a road user would affect other vehicles. In this regard, real-time closed-loop feedback is critical to investigate the interactions of road users with the surrounding environment.

### 2.2. Environments for Intelligent Transportation Systems

In general, the study of human behaviors at given situations is referred to as human factors. The human factors are typically associated with the interaction research of vehicle automation and a road user. In the literature, we find that previous studies consider humans as a part of vehicle automation including combined function automation, limited self-driving automation, and full self-driving automation. The ultimate goal of the research is to eliminate the human errors and uncertainty from automation, and hence, from the viewpoints of drivers, they have mainly focused on user interfaces, acceptance and trust, inattention and distraction, and adaptation to vehicle automation. For instance, many studies of human factors have developed the human–machine interface [12,13,14,31,32]. They include the determination of feedback methods when automated vehicles should notify the warnings and useful messages to the drivers and passengers, such as auditory, tactile, or visual presentation, and timing to transfer information. Some of the previous work investigates human factors for driver’s trust and acceptance of the automated vehicles [15,16,17,18]. They try to identify critical situations and various factors that have a positive or negative effect on driver’s trust. It focuses on how to orchestrate the effects of unfamiliar automation of AVs on humans. Consequently, they not only leave mixed traffic environments out of consideration, but also have no interests in how human drivers will react when human-driven vehicles encounter autonomous vehicles on the road. Our perspective on human factors is significantly different from that of previous human factors studies.

In this paper, we are interested in the methods to find out the unforeseen problems for CAVs interacting with drivers of the CNAVs. Currently, a large number of CAVs do not exist and hence, it is hard to verify the effects they have on mixed traffic and to examine how they interact with humans. There are analysis methods for the factors threatening road safety [33,34,35,36]. In the case of the analysis based on police-reported and driver’s self-reported traffic accidents, the data are likely to be acquired as the police’s technical concern and the interests of the drivers with a highly subjective point of view on the crashes [37]. In addition, it has the issue of under-reporting of crashes and there are no reliable measures to identify the drivers’ behavior in a crash [38]. To minimize the outliers caused by human nature and enhance the reliability of the collected data, it is necessary to accumulate a large number of cases such as traffic accidents. However, not only it will take a long time, but also gathering crash-related data is not easy in the mixed traffic environment. It would be inappropriate to study issues related to the coexistence in such a manner during the period of the transition. To take human behavior into account when modeling vehicular traffic, there is an attempt using actual traffic data collected from real roads. A connected vehicle assessment system (CONVAS), using traffic data called safety pilot model deployment (SPMD), generates realistic vehicular traffic [39]. However, this approach requires a lot of real data to represent different traffic scenarios because the vehicles' movements are always the same. In addition, there are only CNAVs below L2 that are fully controlled by human drivers. There are other studies to model vehicle traffic representing human drivers’ behaviors. The human behavior has complexity depending on a spatio-temporal pattern [40]. Analyzing the behavior pattern and considering the conditions that influence people’s decisions, human dynamics can be reflected to model vehicular traffic and determine routes. A traffic modeling product (i.e., Aimsun Next) developed by Siemens (one of the major signal control manufacturers) is capable of generating various traffic conditions based on either stochastic route choice or dynamic user equilibrium [41]. It can provide a normal traffic condition with steady-state behavior as well as a changing traffic condition with dynamic traffic volumes. However, the two studies may have modeling errors and uncertainties inherent in modeling vehicle movements representing human behavior. It is necessary to re-enact a certain traffic situation on the real road to minimize uncertainty coming from human nature. Therefore, to activate interaction research considering the coexistence, we need to investigate if existing controlled environments help generate reliable datasets of drivers’ intention in complex systems.

In the literature of building controlled ITCPS environments, many studies focus on one aspect among multiple components of ITCPS, depending on the research purpose. Hence, their approach requires a dedicated system performing its functions designated for the specific purpose. For instance, a customized driving simulator has been used to assess the factors affecting the driver’s performance, such as fatigue, disengagement, confusion, and distraction of drivers experiencing the automated driving vehicles [19,20]. When dealing with the uncertainty of drivers, a microscopic traffic simulator has been used to investigate the effect of human reactions as a function of a stop distance at signalized intersections [21]. To investigate the road performance (i.e., a level of stability of traffic streams) as the market penetration rate of V2V communication equipment varies, the models for driving maneuvers (e.g., car-following model) depending on different vehicle types such as AVs, CAVs, NAVs with connectivity, and NAVs without connectivity are added to a traffic simulator [42,43]. They have modeled the human maneuver of NAVs as pre-determined actions of the human drivers on the experiment. However, there may exist a deficiency to entirely represent human nature in the driving model and an inherent modeling error. It is also hard to study how the maneuver of AVs affects the intention and response of human drivers of NAVs. Following such an approach, a distinct system should be newly developed whenever a dedicated system for a specific purpose is needed [19,20,21,22,23]. Occasionally, it should be extended by adding desired functions to existing platforms [38,42].

To address the issues related to the development effort, another approach is to integrate stand-alone simulators through interfaces that allow ways to communicate with each other. In most cases, an integrated environment consisting of the driving and traffic models has been developed [22,25,26,27]. Among them, some attempts do not consider the interactions of the CAVs with human drivers base on V2X communication [22,26,27]. For instance, there are integrated systems that allow a driver to experience realistic driving scenes synchronized with a traffic simulator [22,26]. They consider a human-driven vehicle and autonomously operating vehicles. However, it does not support any standard V2X communication. Only a common description of the road network is exchanged for synchronization through interfaces. In this regard, there is no real interaction between autonomous and human-driven vehicles. Meanwhile, the traffic stream is generated in the macroscopic perspective since they do not deal with a vehicle as an independent object in the systems. It indicates that all vehicles of the system have the same parameter configuration for driving. Hence, their environment cannot support the individual control of simulated vehicles.

Some studies do not consider the automation of NAVs [25,27]. Punzo and Ciuffo have constructed the interactive environment with real-time data exchange between a traffic simulator and a driving simulator that models driver behavior [25]. They do not consider the V2X communication and any automation level of the human vehicle. Jeihani et al. have used the integrated environment to identify the information that influences the driver when determining a route from source to destination [27]. Their integrated system has only human-driven vehicles with L0 of automation without a communication capability. Their vehicles are merely equipped with the navigation system that can exploit real-time traffic information for route guidance but do not interact with the road infrastructure and other vehicles.

To deal with the interactions of CAVs with human drivers, Vokrinek et al. have proposed an integrated environment to quickly develop next-generation technologies for vehicles. It is provided with human-in-the-loop testing and included an artificial intelligence car-following method [44,45]. Although they claim that testing can be performed in various and mixed traffic environments (i.e., simulated vehicles and a human-driven vehicle), there is no cooperation with the transport infrastructure and no support of V2X communication. In addition, it supports one-way traffic to investigate human behaviors and uncertainties. Jin and Lam have also developed an environment to investigate how ITS information affects drivers’ behavior and decision during traveling [46]. Since they adopt an open-loop structure when designing the environment, the information flows only in downstream direction, from the transport infrastructure to the vehicle of a human driver. Human behavior, therefore, does not feed back into surrounding vehicles. Furthermore, they do not consider mixed traffic conditions and V2X communication.

As described above, there have been many attempts to construct a controlled environment. However, they do not support close interactions among individual components for activities of the system. In most existing environments, the requirements discussed in Section 2.1 are not satisfied. An exception is the work reported in [24] to develop an evaluation tool that incorporates traffic, driving, and networking simulators. This work is similar to our interactive ITCPS framework development. However, they focus on specifying interfaces to integrate existing tools, not supporting the detailed design for building a general environment from a macro perspective for the urban ITCPS of coexistence. It is not appropriate to provide an understanding of core components for building an interactive ITCPS environment.

## 3. A Component-Based Interactive ITCPS Framework

We propose an interactive framework for ITCPS, supporting coexistence and interaction with humans. It is challenging to meet all of the diversified requirements representing the different functions and the different levels of the functionality. To address the complexity derived from different levels of the functionality, we begin with identifying and defining the core functions related to ITCPS. Once identified, those core functions can be modeled as components. The behaviors of the components are defined in detail by their functions and activities implementing the component, helping to comprehend the ITCPS’s functional requirements. To accommodate the complexity of many different functions derived from different requirements, we should consider scalability and flexibility when designing an interactive ITCPS framework to be applied to a wide range of transportation applications. In this regard, it should not only be designed to add, modify, and remove any functions required to generate a targeted system at ease, but also intend to simplify the role of components to attain near independence among components.

To provide core services of the ITCPS, we design a new framework with a scalable structure based on component modeling. In software engineering, component-based software development (CBD) is a well-known methodology for reusability, which recombines one or more components required from a set of existing components to build a system [47]. Nevertheless, a standard process for designing components and interfaces covered in the CBD has not been established clearly [48,49,50]. When a designed system is assembled using one or more components, among the existing components, it is rare to find the components to fit perfectly into a designed system. In most of the cases in practice, the CBD accompanies the additional tasks to modify some components and add a new component to be deployed into a system [49]. For these reasons, we intend to take only the common concept of the component-based approach rather than applying the CBD directly to our design.

In this paper, an interactive ITCPS framework refers to a system with components that are characterized by interfaces and behavior models. A component supports one or more services and is defined as a unit independently executing the desired services. Interfaces specify relationships between components [51]. Behavior models are the descriptions of basic activities of the ITCPS, which can represent a set of mathematical functions. The component assembles behavior models since an individual service is implemented as a corresponding behavior model. Therefore, for an interactive ITCPS framework, the component model-based architecture is represented by (1) defining components by decomposing the complex ITCPS into specific services at the macro level, (2) defining interactions among these components, called a component interface, and (3) defining a specific behavior model that effectively supports each component. In this section, the interactive ITCPS framework is described to provide a conceptual understanding of the designed system at an abstract level, independent from the implementation. In the following sections, we discuss the detailed behavior models to implement them.

### 3.1. Conceptual Design

**Figure 1:**
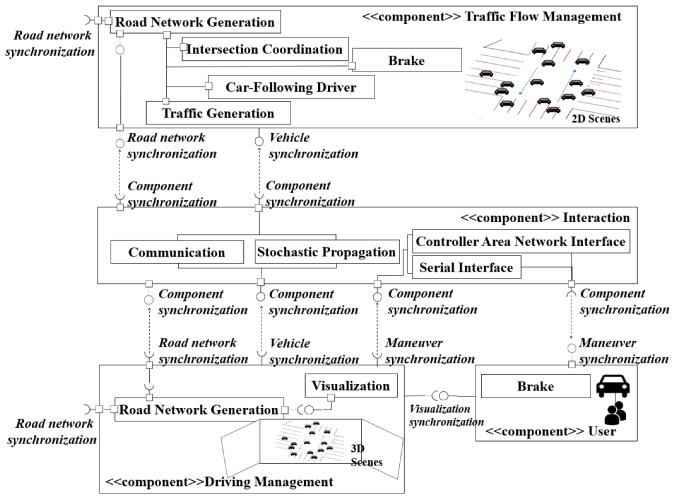
Overall architecture of an interactive intelligent transportation cyber-physical systems (ITCPS) framework.

The overall architecture of the proposed interactive ITCPS framework is presented in Figure 1. There are four components represented by big rectangles. The interfaces between components are represented by a ball-and-socket connection between big rectangles. A ball represents a provided interface and a socket indicates a required interface. Behavior models are represented by rectangles in each component. The dotted arrow in a ball-and-socket connection indicates the relationship between a provided interface and a required interface. A small rectangle connector of a ball-and-socket connection indicates a contact point to directly provide the external contact of the behavior model. To draw a clear understanding of the relationship among the components from Figure 1, an example of the data flow is introduced to provide reference coordinates of a traffic flow management component for a driving management component. In the traffic flow management component, the ball (i.e., an interface marked with a “*road network synchronization*”) provides the reference coordinates for the socket (i.e., an interface marked with a “*road network synchronization*”) of the driving management component. It is delivered through the ball-and-socket (i.e., an interface marked with a “*component synchronization*”) in an interaction component.

#### 3.1.1. Components

The interactive ITCPS framework consists of four components, as shown in Figure 1: A user component, a traffic flow management component, a driving management component, and an interaction component. A user component (UC) is a part for continuous generation of driving information including driver’s behavior. Hence, the UC includes a human driver and a vehicle. A vehicle, being manipulated by a human driver, adopts the real car parts including a steering wheel, acceleration, and deceleration pedals in a vehicle cabin as well as a head-up display (HUD) device. In addition, this vehicle supports L2 of automation including V2V communication and exploits the control information over an in-vehicle network (e.g., a controller area network). Therefore, this vehicle represents a CNAV. Using such a vehicle, the maneuver of the human driver builds a distinct driver model from other drivers.

A traffic flow management component (TFMC) has a role to generate vehicles, manage the vehicular traffic, support intersection coordination, control vehicular movements, and visualize traffic conditions. In detail, the TFMC specifies the number of vehicles on the road, the vehicle’s position, and a travel route. During the transition period, CAVs have over L3 of the automation as mentioned earlier. In fact, since all CAVs managed by the TFMC should obey traffic regulations on a given road when performing a certain driving task, no driver intervention is necessary. The TFMC is responsible for generating two-dimensional (2D) geometry for topological representation of the road layout. The generated vehicle is deployed to a 2D road network.

A driving management component (DMC) builds a 3D driving environment for a human driver to have similar experience as in real driving. The 3D driving environment conveys the visual information to a human driver, including static elements (e.g., driving road types, buildings, walkways, lanes, road signs, and intersections) and dynamic elements (e.g., surrounding vehicles’ movements and traffic lights to change). This periphery of the road may influence user’s behavior and response in direct and indirect ways. Furthermore, the DMC manages the information of the CNAV, which represents the human behavior of the UC.

An interaction component (INTC) has the responsibility of V2V communication of all vehicles, including the CAVs of the TFMC and the CNAV of the UC. The INTC supports the communication environment of a wireless channel quality model and defines the type of messages required for exchanging specific vehicle information. Vehicular networks have specific characteristics such as the high mobility of vehicles and the complicated and multiple propagation paths of transmitting signals. The INTC employs a specific fading model suitable for an urban road to provide V2V communication considering the given position and the speed of vehicles.

#### 3.1.2. Component Interfaces

A component interface determines the external behavior of the component. As shown in Figure 1, the five interfaces focus on the relationship among components at the abstract level, which becomes concrete by the interface definition. We design critical interfaces to perform traffic synchronization for the TFMC and DMC. The interfaces between the TFMC and the DFC specify the synchronization services required for traveling of all types of vehicles (i.e., CAVs and CNAVs). They consist of the synchronization interfaces related to the generation of a road network and CAVs, and the management of vehicular traffic flow as follows:

- Road network synchronization: It helps CAVs and CNAVs operate on the same road by using their coordinates. To build a road network, the road network synchronization interface is used to acquire information of a particular map from the external resource. The TFMC generates a road network that specifies a certain range available for driving, with GPS coordinates. The GPS coordinates of the center point of the road network should be delivered to the DMC to align the center of the road network of the TFMC to that of the DMC. Using this information, the DMC can render the synchronized road network. In this paper, the coordinates representing the center of the given road network is referred to as reference coordinates. This synchronization is performed once when the road network at both sides is constructed.
- Vehicle synchronization: The TFMC is primarily responsible for traffic management including CAVs and CNAVs. In addition, to provide vehicle information for users and manage the CNAVs’ traveling, the traffic flow information should be synchronized with that of the DMC. The information should be transferred to the DMC continuously after CAV is generated.

For interaction between the UC and the DMC, critical interfaces are defined to specify all the services required. These interfaces are to provide the services that include the management of the human maneuvers and the generation of a realistic driving environment as follows:

- Maneuver synchronization: All human maneuvers represent human decisions that may affect the maneuver of the surrounding CAVs during traveling. Using this interface, the driver’s intention should be delivered to the DMC whenever a driver controls individual parts (e.g., steering wheel, brake, accelerator, and gears) of the vehicle cabin. Conversely, to take the appropriate behavior for a certain situation, the driver needs all the surrounding information related to driving. Furthermore, depending on the ADAS (advanced driver assistance systems) applications activated, the UC should deliver a desired warning message to the human driver to ensure driving safety. The maneuver information held by the DMC is used to generate a warning message.
- Visualization synchronization: To create a realistic environment, visualization is critical since it is important to get the reaction of a driver as close to reality as possible. A user of the UC needs to acquire the visual information from a display unit to continuously recognize traffic condition changes including the CNAV, CAVs driving near the CNAV, and the road. This is achieved by providing the driver with the 3D scenes that are continuously rendered by the DMC. Since this synchronization aims to visualize the natural behavior of the vehicle, its performance depends on how well the synchronized data is obtained in time.

Another important interface is a component synchronization interface that is concerned with all four components. A component synchronization interface helps continuously share the information on the current traffic status. The component synchronization aims to help data residing in different components to be consistent with the internal data of a certain component. Hence, this interface allows the vehicles within a given transmission range (radius) of each vehicle to exchange messages. Since the interactive ITCPS framework supports safety-critical applications as primary applications in the C-ITS, the format of the exchange message should conform to the standard SAE J2735 BSM [52]. The BSM contains the vehicles status information such as GPS coordinates, speed, and heading. The transmission time interval of BSM is 100 ms as specified. It also includes the control information for reducing the speed and crossing the intersection, in addition to vehicle status information. Moreover, the component synchronization interface provides the access for control data of in-vehicle network through the direct connection with the CNAV.

### 3.2. Detailed Design

Now, we present the individual behavior models for each component. First, behavior models for the TFMC represent the functions of the road network generation (denoted as a road network generation model), traffic flow generation (denoted as a traffic generation model), vehicle movement (denoted as a car-following driver model), and traffic signal control (denoted as an intersection coordination model). Second, a behavior model for the DMC includes a function of the visualization with an interpolation process (denoted as a visualization model). A behavior model of the road network generation for the TFMC is also involved in the DMC. Third, the INTC is specified by a communication model for V2V communication and a statistical channel model (denoted as a stochastic propagation model). The statistical channel model is very useful for modeling the signal status that is affected by specific geographical deployment, distances, and locations. Finally, the behavior model in the UC deals with a brake system of the CNAV. A brake model represents an AEB (automatic emergency braking) system, which is one of ADAS applications.

#### 3.2.1. A Road Network Generation Model

A road network generation model creates the road network geometry using the information of a given map acquired from OpenStreetMap (OSM), which provides the geographic data of the whole world [53]. The map data consist of a set of 2D information representing a road network of GPS coordinates, the name of a certain street, the classification of objects such as subways, roads, and hospitals. A road network generation model first transforms such 2D information to 3D information with height by using one of 3D modeling software [54]. After this process, to identify individual objects on the road network, we use three types of coordinates: GPS coordinates, UTM (universal transverse mercator coordinate system) coordinates, and local coordinates. The GPS coordinates of individual objects are provided by the map data. Over the traffic synchronization, the vehicular location data between the TFMC and the DFC are explicitly synchronized using the GPS coordinates of the BSM. The TFMC and the DMC internally use the UMT coordinates to specify the location of each object on their road networks. The local coordinates are used to continuously render various objects. By using the library of the coordinate system transformation, the GPS coordinates of an object are converted into the UTM coordinates whenever the raw data of the road network including individual objects are loaded [55]. The local coordinates are transformed by using both the UTM coordinates and the reference coordinates. The local coordinates, denoted as $\left(x_{l},\;y_{l}\right)$, of an object are given by:

(1) $$\left[\begin{array}{c} x_{l}\\ y_{l} \end{array}\right]=\left[\begin{array}{c} \left(x_{UTM}-x_{r}\right)\times \beta \\ \left(y_{UTM}-y_{r}\right)\times \beta \end{array}\right],$$

where $x_{UTM}$ and $y_{UTM}$ are the transformed UTM coordinates with the x-coordinate and y-coordinate of a vehicle, respectively, and β indicates the scale factor (meters per pixel) and is set to 10. $x_{r}$ and $y_{r}$ are the reference coordinates and are set to be 450,939.91 and 3,950,659.43, respectively, in this paper. Since it is not necessary to convert the height of the object when the objects are identified and rendered, it is not used for transformation. In addition, an existing signalized intersection among road surroundings obtained from OSM is transformed into an intersection without traffic lights in our road network and the lane for each direction is configured with three lanes.

#### 3.2.2. A Traffic Generation Model

In our framework, the generation rate of CAVs follows the uniform distribution and each travel route is specified either randomly or by the shortest route. The speeds of vehicles are limited by the maximum road speed. It is variable with the default value of 60 km/h in our road network. To build an acceptable ITCPS environment, the number of traffic objects representing the vehicles should be generated similar to the traffic volume observed on the actual road. We describe how to determine the maximum number of vehicles available for all roads in our environment. A maximum traffic volume acceptable for our road network is referred to as the road capacity, which is commonly defined as a maximum hourly rate of vehicles (vehicles/hour) that are expected to pass through a given road section. A design hourly volume (DHV), which is the basis of road design, is commonly used to determine the road capacity and the number of lanes [56]. While applying to road design, the DHV is calculated with the product of annual average daily traffic (AADT) and a design hour factor. However, due to the change in the configuration such as the number of lanes and the un-signalized intersection, it is not appropriate to use the actual traffic volume survey of the AADT for our environment. In fact, the road capacity of our road network is bound to be highly influenced by the road section of an un-signalized intersection which may lead to congestion and long delays. Therefore, we do not use the DHV to determine the road capacity, but rather the analysis of the un-signalized intersection capacity. In this paper, a formula of the Berry–Gandhi method, which estimates a lane capacity (vehicles/lane/hour) for a signalized intersection, is modified to apply to our specific case with an un-signalized intersection [57]. The details are discussed in the following.

The road capacity is characterized by two parameters: The sum of the critical-lane volumes (CLVs) and the number of lanes. A critical lane is a lane with the most intense traffic during a green signal [58]. We utilize the sum of the CLVs, denoted as ψ, to estimate the maximum capacity for all lanes. In our road network, a vehicle can cross an un-signalized intersection without any delay and collision by autonomous intersection coordination, which will be explained in Section 3.2.4 in detail. On the vehicle side, it is as if the vehicle’s movement is always permitted by a green signal for its driving direction. For this reason, it is assumed that vehicles cross the intersection without delay, regardless of any driving direction. Therefore, in our network, the CLV can be determined using the planned green signal phase under the assumption that there is only a green signal even though it is an un-signalized intersection. The individual signal distributed to each direction of vehicles’ movement within a signal cycle is called phase. The CLV for one hour corresponds to the maximum value of flow rates per lane (vehicles/lane/hour) that indicates the maximum number of vehicles moved in one lane for one hour, assuming the signal to be only green. Therefore, the sum of the CLVs can be greater than the actual traffic volume of the road network.

**Figure 2:**
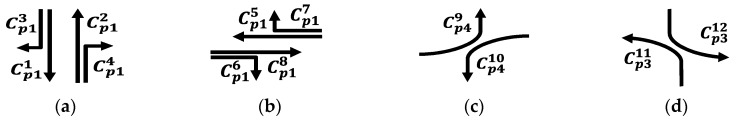
Movements of vehicles in four phase signals with the green light at a four-way intersection and a flow rate, $C_{pi}^{k}$, where i and k are the identifiers of each phase and flow, respectively. (**a**) Phase 1; (**b**) Phase 2; (**c**) Phase 3; (**d**) Phase 4.

Our road network has a four-way un-signalized intersection with three lanes for each way. In this regard, we can assume that there are four green-phase signals and the lane flows for a green signal at the un-signalized intersection as shown in Figure 2. The non-conflicting flows, which go straight across the intersection, are put into the same phase. At the same time, the non-conflicting right turn flows are also grouped into this same phase. Therefore, in Phase 1 and Phase 2, it is designed that flows of the two lanes for each way move together. The left-turn flows of the other lane cross the intersection together for Phase 3 and Phase 4. All vehicles in our framework are designed to move along with virtual four-phase signals. A flow volume (FV) represents the number of vehicles that can be moved in one lane for each phase (vehicles/lane/phase). The FV is denoted as $C_{pi}^{k}$, where k indicates an identifier for each flow and i is a phase identifier as shown in Figure 2.

With using FVs, the CLV for Phase i, denoted as $CLV_{phase}^{i}$ (vehicles/lane/phase), representing the maximum amount of the traffic flows to move during a given phase i, is defined as:

(2) $$CLV_{phase}^{i}=\max\left\{0,C_{pi}^{k}\right\},$$

where i is the identifier of the phase within the range of 1 and 4 phases and k is the identifier of the flow, which belongs to the phase i. Note that k is within the range of 1 and 12 flows. For one hour, each phase’s CLV $\psi_{i}$ (vehicles/lane/phase/hour) is yielded using $CLV_{phase}^{i}$ as follows:

(3) $$\psi_{i}=m\times CLV_{phase}^{i},$$

where m is the number of cycles for one hour and $CLV_{phase}^{i}$ presents the CLV of Phase i. Using the Berry–Gandhi method, we can obtain a total of CLVs ψ (vehicles/lane/hour) as $\sum_{i=1}^{n}\psi_{i},$ where n is the number of phases. Since the road capacity χ is the maximum traffic flow rate in a given road network using all available lanes as mentioned above, it can be defined as the sum of CLVs for all lanes. Hence, the road capacity χ (vehicles/hour) can be described by the following equation:

(4) $$\chi =\psi \times L_{i}=\sum_{i=1}^{n}\psi_{i}\times L_{i},$$

where $\psi_{i}$ is the critical-lane volume per phase for one hour, i is a phase identifier, n is the number of phases, which is four as shown in Figure 2, and $L_{i}$ is the number of lanes for each phase.

Furthermore, in the Berry–Gandhi method, since the lane capacity (vehicles/lane/hour) is computed under the condition where the signal is always green and there is no transit delay, the lane capacity can be defined as the total of the CLVs ψ (vehicles/lane/hour). The average headway time $\bar{h}$ is the constant value, which is measured as the time between vehicles passing the reference line of a stable platoon during a green signal at the intersection. It is almost equal to an average starting time delay $\bar{D}$ elapsing from the beginning of a green signal to the instant the rear wheels of the first vehicle cross the reference line. In our environment, the length of the signal cycle $T_{C}$ is the same as the length of the green signal $T_{G}$ because there is only the green signal. To compute the lane capacity ψ in vehicles per hour, the Berry–Gandhi method is modified to the following equation:

(5) $$\psi =\frac{3600\times \left(T_{G}-\bar{D}+\bar{h}\right)}{T_{C}\times \bar{h}}\approx \frac{3600}{\bar{h}}.$$

Since the number of lanes for each phase is different, a road capacity for our road network is estimated as follows:

(6) $$\psi \times L_{min}\ll \chi <\psi \times L_{max},$$

where $L_{max}$ and $L_{min}$ are the maximum number and the minimum number of the lanes allowed for one phase, respectively. The above equation is based on the assumption that the go-straight movement volume is the same as its corresponding right-turn movement volume in Phase 1 and Phase 2. It is also assumed that the movements of the other lane can be sufficiently accommodated since the bigger volume is determined as a CLV between traffic volumes of each phase. From Equation (6), the road capacity is computed to be within the range of 3829 v/h at $L_{min}=2$ and 7659 v/h at $L_{max}=4$ when we take the value of the average headway time as 1.88 s [59].

#### 3.2.3. A Car-Following Driver Model

A microscopic model describes the behavior of each vehicle interacting with surrounding vehicles. The physical propagation of traffic flows is estimated by the dynamic interactions among drivers, vehicles, and roads. In modeling the vehicular traffic flow in the microscopic scale, all of the individual vehicles should be independently treated on the roads. Therefore, representing microscopic flow variables of the headways of distance and time, the maximum speed, the maximum acceleration, and the size of acceleration is critical [60]. Such variables consequently make the vehicle trajectory, which has a list of the positions of the vehicle over time. Especially, the CAVs travel along the designated path according to driver behavior models including car-following, lane changing, and safety-distance keeping. We use the car-following driver model with safety-distance keeping to support the driving function of longitudinal control and navigation. During driving, the flow variables of the speeds and the size of the acceleration and deceleration of the individual CAVs are determined by a car-following driver model. In the case of CAVs, the headway is kept to the minimum time since it is only governed by the automation of a CAV. The distance headway is determined depending on the time headway of the CAV and the speed of the leading CAV [60]. While the movements of CAVs are highly affected by an intersection with traffic lights of the designed road network, it is not necessary to consider its effect on the movements of the CAVs, since the interactive ITCPS framework has only an un-signalized intersection.

Car-following driver models have received considerable interest in the transport domain and there are many studies since the 1950s. Among them, we apply the simple and powerful Newell’s model to present the behavior of CAVs in the ITCPS framework, which follows the same route of the precedent vehicle with a given headway time [61]. This model does not require a large amount of computation to create the movement of the following vehicle since it uses the movement of the precedent vehicle. A Newell model assumes that the time-space trajectory of the ith vehicle is very similar to that of the i − 1th vehicle, which is a preceding vehicle of the ith vehicle, when several vehicles are driving in a chain. The positional relationship between the two vehicles is given by:

(7) $$p_{i}\left(t+\tau_{i}\right)=p_{i-1}\left(t\right)-d_{i},$$

where $p_{i}\left(t\right)$ is the position of the ith vehicle at the time t, $\tau_{i}$ is the time offset, and $d_{i}$ is the inter-vehicle distance offset. Equation (7) is formulated such that a following vehicle after the given time of $\tau_{i}$ is expected to be at the position that is $d_{i}$ away from the position of the preceding vehicle. This value of $d_{i}$ occurs as delay because the driver of the following vehicle determines its maneuver according to the maneuver of the preceding vehicle as mentioned above. The time offset depends on the designated safety distance. In the case of our ITCPS framework, since CAVs are not controlled by human drivers, the variables for the perception and reaction of the human do not affect this driver model. Therefore, for our framework, the car-following driver model of CAVs is given as:

(8) $$p_{i}\left(t+\tau_{i}\right)=p_{i-1}\left(t\right).$$

Besides our car-following driver model of Equation (8), we can apply any type of traffic model belonging to a microscopic traffic model. For example, depending on the needs of the user while driving, a traffic model takes into account personal preferences affecting people’s behaviors such as fuel consumption and traveling time [40]. We can adopt either a new traffic model that takes into account variables such as driver distraction and reaction delay in driving [62] or a traffic model that reproduces the movement of the vehicle by using the actual driving data [63].

#### 3.2.4. Intersection Coordination Model

In urban roads, many vehicles meet at an intersection that may cause inefficient traffic congestion. To address the issues derived from an intersection with the mixed traffic situation, an un-signalized intersection is designed in the road network. However, we cannot avoid traffic jams and collisions if an intersection coordination strategy for CAVs and CNAVs does not support them passing the un-signalized intersection safely. A simple way to ensure the intersection safety is that a vehicle approaching the intersection is given a right-of-way to cross the un-signalized intersection. The method of determining the right-of-way is referred to as an intersection protocol. The intersection protocol is performed by either a centralized or a distributed method. The centralized method is to perform the determination of the right-of-way in the intersection management systems connected to roadside units. It collects the necessary information using vehicle-to-infrastructure (V2I) communication and informs all vehicles approaching the intersection of its decision. In the distributed method, each individual vehicle makes a decision whether or not to cross the intersection. The decision-making depends only on V2V communication without any centralized system. A vehicle approaching the intersection determines its strategy based on the information collected from the BSMs it received. In our framework, we use one of distributed methods for vehicles to cross the intersection safely [64].

In the intersection coordination model, an un-signalized intersection is divided into small rectangular regions, called cells. All vehicles determine the path consisting of the cells of the intersection before crossing the intersection and then transmit a list of all the cells on the path to neighboring vehicles. Before a vehicle enters the intersection, the vehicle determines its crossing strategy by comparing its path with the received paths. When a vehicle enters an intersection while maintaining its current velocity, it is possible to meet another vehicle at the same cell at a certain time. In this case, to avoid a crash, each vehicle obtains the priority of the right-of-way determined by the first-come first-served principle using the vehicles’ arrival time at the intersection. The CAVs adjust their velocities according to the determined priority [64]. However, in the case of CNAV, the driver is not guided to adjust the velocity of the vehicle. If the intersection coordination model determines to assign the right-of-way with the highest priority to a CNAV before it crosses the intersection, the vehicle’s HUD device signals the driver to cross the intersection. It means that the driver should keep the current speed. If not, a stop sign is displayed on the HUD device of the CNAV. In this regard, the intersection crossing assistance service (ICAS) includes such a visual warning service to inform the CNAV.

**Figure 3:**
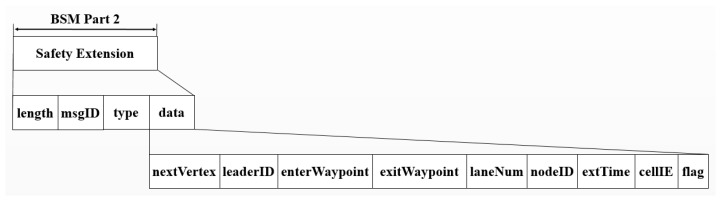
Design of the Society of Automotive Engineers (SAE) J7235 basic safety message Part 2 containing the information for performing an intersection protocol.

To perform the intersection protocol, the intersection coordination model utilizes the information that is encoded into the data element in the SAE J2735 BSM Part 2 as shown in Figure 3. We designed the data element of the SAE J2735 BSM Part 2, which is described in detail in Section 3.2.7. An element of *nextVertex* contains the identifier of the intersection that a leader vehicle is approaching. The leader vehicle refers to a vehicle that is at the forefront of preceding vehicles in a particular lane where a given vehicle is driving. An element of *leaderID* has the identifier of the leader vehicle, which is required because the velocity of a given vehicle is calculated based on that of the leader. The waypoints of the intersection that a given vehicle enters and comes out are presented as GPS coordinates in the elements of *enterWaypoint* and *exitWaypoint*, respectively. The lane and the road segment where a given vehicle travels are presented in the elements of *laneNum* and *nodeID*, respectively. A list of all cells on the path crossing the intersection is encoded into the element of *cellIE*. The element of *flag* contains the information whether or not pedestrians or obstacles exist at the intersection.

#### 3.2.5. A Visualization Model

Since the visualization synchronization is carried out over V2V communication, the visualization uses discrete-time data. The UC suffers from missing values until the next synchronization time. If the visual driving environment is updated to keep pace with that interval, it causes visual flickering of virtual vehicles, resulting in a significantly degraded driver’s recognition of the reality. To address this problem, we utilize the refresh rate available on a display device. For example, in the case of the refresh rate γ of 50 Hz, new driving scenes are generated five times within the synchronization interval that corresponds to the transmission time interval (i.e., 100 ms) of a BSM.

A visualization model continually generates a series of the virtual driving scenes for the CNAV by a view interpolation process [65,66]. To achieve the interpolation showing the surrounding vehicle’s movements, the visualization model uses the displacement of the surrounding CAV to estimate its next position (i.e., the coordinates). Displacement is the difference in the previous and next positions updated by two successive BSMs of the vehicle. The estimated position of the vehicle may accumulate the difference from the actual vehicle position until it is calibrated at every 100 ms. During the synchronization interval, a threshold and a weight moving average (WMA) method are used. The visualization model performs three steps within the synchronization interval: The acquisition of the coordinates of a vehicle just after the synchronization, the update on displacement for a unit of time, and the determination of the estimated coordinates of a vehicle. The visualization model first examines a residual generated by comparing the estimated coordinates ($x_{e},\;y_{e})$ of the visualization model with the actual vehicle coordinates ($x_{bsm},\;y_{bsm})$ of the BSM received. As mentioned above, the local coordinates, converted from the GPS coordinates, are used for all processes to estimate the position. The residual, denoted as ε, is given as:

(9) $$\varepsilon =\left|x_{bsm}\left(t\right)-x_{e}\left(t-1\right)\right|+\left|y_{bsm}\left(t\right)-y_{e}\left(t-1\right)\right|,$$

where $x_{bsm}\left(t\right)$ and $y_{bsm}\left(t\right)$ are the converted x-coordinate and y-coordinate of the vehicle in the BSM received at the time t, respectively, and $x_{e}\left(t-1\right)$ and $y_{e}\left(t-1\right)$ represent the estimated x and y-coordinates at the time t−1, respectively. The time t−1 indicates the latest time at which the coordinates are estimated before the new BSM is received at time t. The visualization model determines if the residual is higher than a given threshold $\eta_{\varepsilon }$. If the residual is over the threshold, the estimated coordinates are immediately updated using the actual vehicle coordinates. If not, its estimated coordinates at the time t is calculated based on the actual vehicle coordinates by using WMA as follows:

(10) $$\begin{array}{c} \left(x_{e}(t),\;y_{e}(t)\right) \end{array}=\{\begin{array}{ccc} (x_{bsm}\left(t\right), & y_{bsm}\left(t)\right), & if\;\varepsilon \geq \eta_{\varepsilon }\\ (x_{bsm}\left(t\right)\times \omega +x_{e}\left(t-1\right)\times \left(1-\omega \right), & y_{bsm}\left(t\right)\times \omega +y_{e}\left(t-1\right)\times \left(1-\omega )\right), & otherwise \end{array}$$

where ω is a weighted value in the range of 0 to 1.

After updating the estimated coordinates at the time t, the displacement, which is denoted as ($\Delta x_{e},\;\Delta y_{e},\;\Delta h_{e})$ representing the direction and the length between two points, is calculated using the following equation:

(11) $$\left[\begin{array}{c} \Delta x_{e}\\ \Delta y_{e}\\ \Delta h_{e} \end{array}\right]=\left[\begin{array}{c} \frac{x_{e}\left(t\right)-x_{e}\left(t-1\right)}{\delta_{time}}\\ \frac{y_{e}\left(t\right)-y_{e}\left(t-1\right)}{\delta_{time}}\\ \frac{h_{bsm}\left(t\right)-h_{e}\left(t-1\right)}{\delta_{time}} \end{array}\right],$$

where $\delta_{time}$ indicates the transmission time interval of the BSM, and $h_{bsm}\left(t\right)$ and $h_{e}\left(t-1\right)$ are the actual heading of the BSM received at time t and the estimated heading of the visualization model at time t−1, respectively. During the time unit Δt depending on the refresh rate γ, the vehicle’s heading and the total amount of movement of the vehicle are expressed as:

(12) $$\left[\begin{array}{c} \Delta x_{e}\left(t\right)\\ \Delta y_{e}\left(t\right)\\ \Delta h_{e}\left(t\right) \end{array}\right]=\left[\begin{array}{c} \Delta x_{e}\times \Delta t\\ \Delta y_{e}\times \Delta t\\ \left(h_{bsm}\left(t\right)+\Delta h_{e}\right)\times \Delta t \end{array}\right],$$

where Δt is the time interval given as the reciprocal of the refresh rate γ. In this regard, time t−1 is defined as time t−Δt. Using Equations (10) and (12), the estimated position and heading at the time t+Δt are generated by the following equation:

(13) $$\left[\begin{array}{c} x_{e}\left(t+\Delta t\right)\\ y_{e}\left(t+\Delta t\right)\\ h_{e}\left(t+\Delta t\right) \end{array}\right]=\left[\begin{array}{c} x_{e}\left(t\right)+\cos(\Delta h_{e}\left(t\right)+\frac{\pi }{4}\times \sqrt{\Delta x_{e}{\left(t\right)}^{2}+\Delta y_{e}{\left(t\right)}^{2}}\\ y_{e}\left(t\right)+\sin(\Delta h_{e}\left(t\right)+\frac{\pi }{4}\times \sqrt{\Delta x_{e}{\left(t\right)}^{2}+\Delta y_{e}{\left(t\right)}^{2}}\\ \Delta h_{e}\left(t\right) \end{array}\right].$$

These estimated values are used continuously to show the movements of surrounding vehicles. In this paper, the weight ω, the refresh rate γ, and threshold $\eta_{\varepsilon }$ are set to 0.7, 60 Hz, and 3 m, respectively.

#### 3.2.6. A Stochastic Propagation Model

A Rayleigh fading model is commonly used for an urban area with static obstacles such as many buildings and roadside trees along the roads. However, the mobility of the vehicle increases the uncertainty of a wireless vehicular communication environment. It causes the frequency shift due to the Doppler effect, the location variations of the transceiver used for communication, and the metal of the exterior material of the vehicle. Such an environment tends to have much more severe conditions than a normal outdoor environment. The Nakagami-m fading model is proposed because the scattering of waves in a certain condition does not coincide with the Rayleigh fading model [67,68]. It can be adapted to various kinds of fading models according to given parameters. We apply this model to the design of the V2V communication environment and a reception threshold to determine if a packet transmitted between the vehicles is successfully received. A reception threshold, denoted as $\eta_{\gamma }$ used in our ITCPS framework, is given as the following:

(14) $$\eta_{\gamma }=e^{-\left(\delta \times m_{f}\right)}\times \overset{m}{\underset{i=1}{\sum }}\frac{{\left(\delta \times m_{f}\right)}^{i-1}}{\left(i-1\right)!},$$

(15) $$\delta ={\left(\frac{d_{s,r}}{d_{tr}}\right)}^{2},$$

(16) $$d_{s,r}=\sqrt{{\left(x_{r}-x_{s}\right)}^{2}+{\left(y_{r}-y_{s}\right)}^{2}},$$

where $m_{f}$ is a m-factor used for the Nakagami-m model, $d_{s,r}$ is a distance between the receiver node r and the sender node s, and $d_{tr}$ is the transmission range of the vehicle for V2V communication. The value of $m_{f}$ has a range between 0.5 to 2 with a default value of 1 and $\delta_{tr}$ is set to 300 m in this paper.

#### 3.2.7. A Communication Model

**Figure 4:**
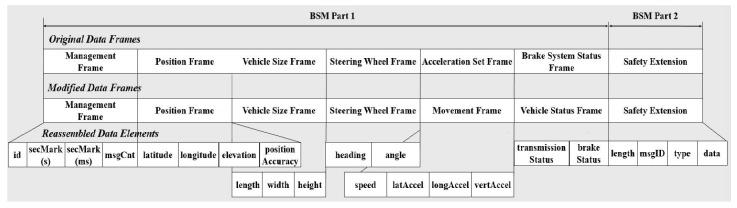
Modified structure of a SAE J2735 basic safety message for vehicle-to-vehicle communication.

To support V2V communication, the information of the vehicles on the road is exchanged in the format of the BSM specified in SAE J2735. The SAE J2735 BSM is divided into two parts. It is mandatory for a communication model to send BSM Part 1, which is a core part containing the vehicle status. Depending on applications, every element defined by users can be added to BSM Part 2. Therefore, the additional elements are specified in the BSM Part 2 to carry the necessary information for specific applications such as the ICAS while the BSM Part 1 is used for road safety. The communication model transmits BSMs at every 100 ms to the neighboring vehicles within the transmission range of a given vehicle. In order to facilitate the data processing in the communication model, the structure of the original data frames of the SAE J2735 BSM is modified as shown in Figure 4. It shows the elements belonging to modified data frames used in the communication model.

As shown in Figure 4, four new elements in the BSM Part 2 are defined for the safety extension frame. The *type* element has one of the values *Warning_Serivce* and *Intersection_Service*. In the case of the visual warning service, the *msgID* element has one of the values *WARNING_SLOWDOWN* and *WARNING_COLLISION* and the *data* element contains a specified warning message. If the value of the *type* element is given as *Intersection_Service*, the *msgID* element contains the identifier of the specific intersection protocol. To perform the intersection coordination model, the *data* element has additional information as shown in Figure 3. In BSM Part 1, the *transmissionStatus* element is used to present either manual or automatic transmission of a given vehicle. We change the role of the *transmissionStatus* element of the vehicle status frame to easily distinguish a receiving node from a transmitting node. In connection with the modified data frames of Figure 4, every element used for the communication model is summarized in Table 1.

#### 3.2.8. A Brake Model

In the near future, it will be mandatory to install an AEB system on all new vehicles operating in the US and Europe [69,70]. Since the interactive ITCPS is designed to support the CNAVs with up to L2 of the automation, we use the AEB system. The L2 of the automation is achieved when (1) two representative functions can be combined, or (2) two ADAS services can be independently performed. In this paper, a brake model is designed to perform combined functions of the forward-collision warning and the braking of an AEB system. Our braking model is designed to detect critical situations based on V2V communication without forward-looking sensors such as RADAR (radio detection and ranging), LiDAR (light detection and ranging), and camera. The brake model consists of three phases: Forward collision warning, deceleration control, and full braking control. A forward-collision warning message aims to provide the driver with a chance to control the CNAV’s velocity by informing the driver of the risk in advance. If the driver ignores this warning message, the velocity control is governed automatically following the phases of deceleration control and full braking in our brake model. The time of braking activation is based on time-to-collision (TTC), which means the time remaining until the collision with the preceding vehicle [71,72]. To calculate this value, the BSMs of the preceding vehicle should be continuously analyzed. The TTC, denoted as $\delta_{ttc}$, of the vehicle is estimated as follows:

(17) $$\delta_{ttc}=\frac{p_{i-1}\left(t\right)-p_{i}\left(t\right)-l_{i}}{v_{i}\left(t\right)-v_{i-1}\left(t\right)},$$

where $v_{i}\left(t\right)$ and $p_{i}\left(t\right)$ indicate the velocity and the position of a given vehicle, denoted as i, at the time t, respectively, i−1 indicates the preceding vehicle of the vehicle i, and $l_{i}$ is the length of the vehicle i. When the TTC value reaches a given threshold $\eta_{w}$ to determine forward-collision warning, a warning message (i.e., potential forward collision is detected) is immediately delivered to a driver to reduce the CNAV’s velocity. If the driver does not comply with this instruction, the deceleration control starts with a certain braking force when the TTC value reaches a given threshold $\eta_{d}$. The braking force for deceleration control is set to 0.4 g. When the TTC value drops below a given threshold $\eta_{f}$, the full braking control is started by pressing the brake with a braking force of 1.0 g to stop the vehicle before the collision. Those thresholds ($\eta_{w}$, $\eta_{d},$ and $\eta_{f}$) are set to 2.6 s, 1.6 s, and 0.6 s, respectively, the same TTC values used in the commercial vehicles [73].

### 3.3. Human and Hardware-in-the-Loop System

**Figure 5:**
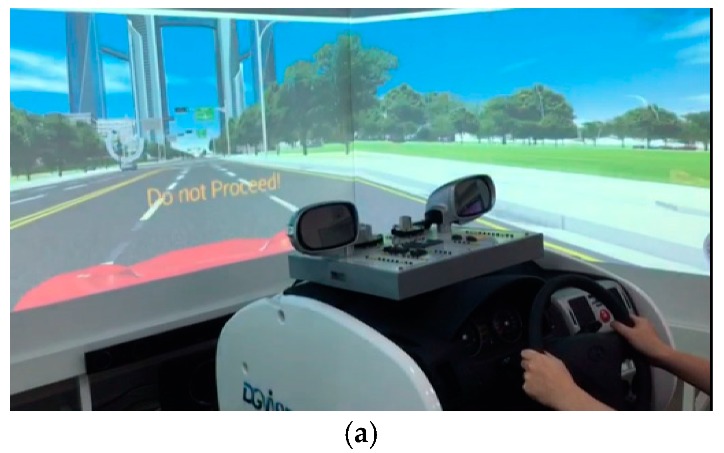

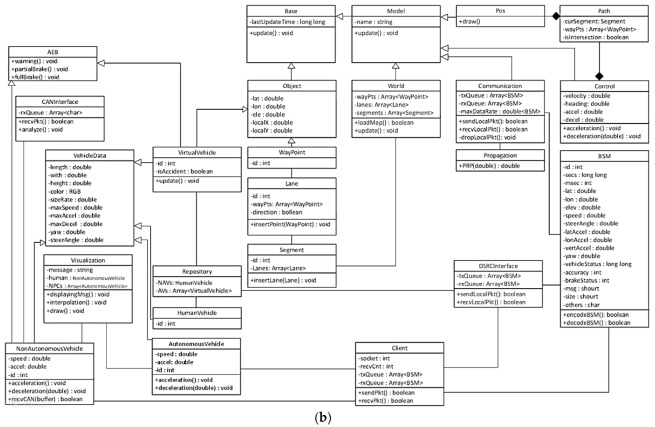
Human and hardware-in-the-loop system (H2iLS) for the interactive ITCPS framework: (**a**) Snapshot of the H2iLS; (**b**) a class diagram for the H2iLS.

An interactive ITCPS framework specifies an integrated complex system that requires various components to be tightly coupled in a realistic manner. To support human interactions, we construct the proposed framework as a form of a human and hardware-in-the-loop system (H2iLS). The H2iLS is emerged as a powerful and effective tool for situations that are difficult to investigate in real life because of complex relationships among various components and the uncertainty of individual human behaviors. As shown in Figure 5a, the physical environment of the interactive ITCPS consists of an audiovisual device connected server-class hardware, and a human driver and a customized vehicle cabin of the UC. The cyber environment is constructed by three components of the TFMC, the DMC, and the INTC, and operates on the server-class hardware. The cyber environment is implemented with a class diagram as shown in Figure 5b. Moreover, the H2iLS has an additional loop for human feedback derived from a human driver of the UC. The server-class hardware consists of Intel Xeon E5 2670-2.6 GHz and 96 GB RAM running Linux and Windows operating systems.

Note that the current prototype implementation has only one customized vehicle cabin directly connected to the server-class hardware using serial and CAN (controller area network) ports. However, our H2iLS can be extended to connect more cabins to the server-class hardware through Ethernet. Therefore, multiple drivers can experience the same traffic scenario with their manipulation of the customized vehicle cabins at the same time. The TFMC is developed from the GrooveNet model based on C++ [74]. The DMC is implemented by adding our behavior models to the reusable modules of OpenDS based on JAVA [75]. A road network corresponding to a specific area of Pittsburg is selected and all roads are modified to have two-way with six lanes. In addition, a four-legged intersection without traffic signals is included in our road network. The transmission range of vehicles for V2V communication is set to the default value of 300 m as discussed before.

## 4. Performance Evaluation

It is necessary to assess how well the implemented system behaves according to the design of the interactive ITCPS framework. In this regard, testing and evaluation of the implemented system to ensure the functionality required for the H2iLS is critical for the completion of the framework design. We use a set of parameters to evaluate its performance, in terms of practicality of traffic management, interactions, and real-time processing. Furthermore, we assess the performance of the AEB service in terms of the functionality of vehicle control.

In general, a serious drawback in microscopic traffic simulation is high computation time because it models every object independently. This feature makes it difficult to support real-time management and processing in response to a certain situation when it deals with lots of vehicle objects simultaneously. The number of vehicles on the road at a given point in time represents a level of system load dealing with vehicular objects. It becomes heavier as the traffic volume increases. To examine the robustness and reliability of the H2iLS in a saturated situation, we evaluate the performance with increasing traffic volume until it reaches the maximum number of vehicles. In Section 3.2.2, the maximum number of vehicles acceptable on the road network is formulated as the road capacity χ. In calculating the road capacity, note that our road network has a different number of lanes at each phase (e.g., either two or four lanes per phase). Also, note that the road capacity is estimated to be greater than the actual traffic volume because the road capacity is based on the number of lanes and CLVs. Hence, we use an approximation of the road capacity by taking the weighted average of those values. We give more weight (α = 0.7) to the number of vehicles for the $L_{max}$ than that for $L_{min}$ in order to generate the saturated situation in which the number of vehicles may exceed the road capacity of the designed road network. For the evaluation, using Equation (6), the road capacity is calculated as:

(18) $$\chi ≒\left(1-\alpha \right)\times \left(\psi \times L_{min}\right)+\alpha \times \left(\psi \times L_{max}\right).$$

Given the time headway of 1.88 s, in our experiments, the road may be saturated when the H2iLS generates up to 540 vehicles moving along predefined paths without collision for 5 min.

First, in our H2iLS, traffic management is expected to improve driving safety and reduce vehicles’ travel time by helping vehicles cross the intersection without collisions by the V2V-based intersection protocol. This function should be carried out under any condition (e.g., heavy load on the system) while vehicles travel in our road network. To examine functional performance capability, we examine both mean intersection-crossing time (MICT) and mean travel time (MTT) measured at the same time as a function of the traffic volume. They represent the consequences of the traffic management function. The intersection-crossing time is measured as the time it takes for a vehicle to cross the intersection after entering the intersection. The travel time is defined to be the time it takes for a vehicle to travel from a given source point to a destination point. Second, to support efficient traffic management, it requires up-to-date traffic information collected through V2V communication in the H2iLS. Since the performance of our framework is highly affected by the interactions among the components, it is essential to perform the process of periodically conveying the vehicle status information in time. To examine how well the H2iLS fulfills V2V communication, a mean transmission time interval (MTTI) of the BSMs is measured as a function of the traffic volume. Third, to evaluate the real-time performance, mean processing delay (MPD) of the H2iLS is measured as the traffic volume increases. The processing delay measures a time interval between the time to start performing a given function and the time to finish it within one processing cycle. In the case of the visualization model, it is required to execute it within the processing time of 16.6 ms because its processing depends on the refresh rate of 60 Hz. Its processing time is the minimum among the behavior models. Therefore, we design a processing cycle of a given function as 16 ms and data variables are synchronized at every 16 ms.

Consequently, the functions involved in the road network generation, car-following driver, traffic generation with routing and path tracking, communication, stochastic propagation, and intersection coordination models have been evaluated. In this paper, it is assumed that while achieving the periodic transmission of BSMs at a given interval (i.e., 100 ms), our framework is capable of processing and managing the desired services in real-time if the MPD stays below the time (i.e., 16 ms) required for performing a given function. Our experiments related to MTT, MICT, MTTI, and MPD are conducted for five minutes with the scenario that one vehicle is generated uniformly at every one second at all lanes. The results are measured from 100 trials of the experiment. Each vehicle in the experiments selects randomly the source and destination points. The maximum speed of vehicles in an urban city is limited to 72 km/h and a transmission range of each vehicle is set to 300 m. The experiment is conducted under the condition where a CNAV should comply strictly with the warning information and traffic rules. In that case, no collision occurs at the intersection.

**Figure 6:**
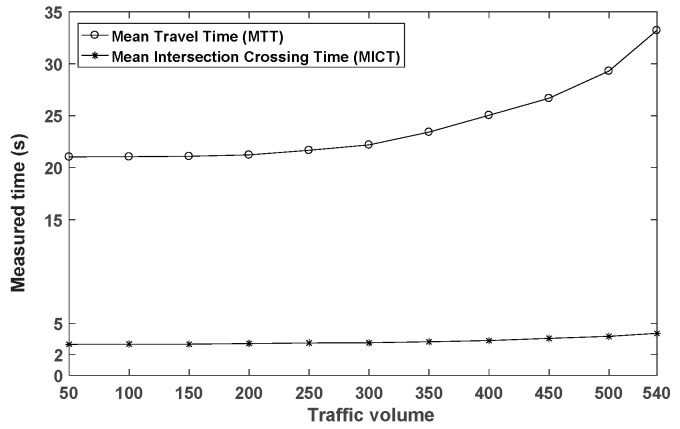
Performance of the mean travel time and the mean intersection crossing time as a function of traffic volumes.

Figure 6 shows that the MICT increases only slightly even though the traffic volume reaches the maximum number of vehicles. From the analysis of the fit function (simple regression), the trend line of the MICT has 0.001918 of the slope as a function of traffic volumes (i.e., y=0.00191765x+2.727767681 with the adjusted $R^{2}$ of 0.808654). The traffic volume does not affect the performance of vehicles crossing the intersection. Figure 6 also presents the trend line with the increase of the MTT that is given by y=0.021863x+17.63285 with the adjusted $R^{2}$ of 0.7676593. From Figure 6, we conclude that the MICT does not affect the vehicles’ MTT, which simply increases because the vehicles could not speed up as the traffic volume increases. In addition, we have shown that our H2iLS has a service capability to perform the designed traffic management functions since the trend lines of the MICT and MTTI are represented by a linear equation with few changes in slope. It indicates that the performance is not affected by traffic volume.

Table 2 presents the MTTI for V2V communication and the MPD. After sending individual BSMs, the H2iLS starts to build the new BSMs and repeatedly sends each BSM to corresponding neighbors at every 100 ms. In the case of the MTTI, the linear regression equation is given as y=0.0006x+100.0058 with the adjusted $R^{2}$ of 0.925942. It turns out that the maximum error is 418.8 μs in the worst case since the MTTI exists in the range of 100.3872 ± 0.03158 with the 95% confidence interval when the traffic volume is 540. Consequently, the H2iLS periodically performs the transmission of the BSMs at every 100 ms, irrespective of the number of vehicles. In Table 2, the MPD is measured as the total amount of time spent to perform designated tasks including data synchronization for the duration of 5 min. In the H2iLS, the behavior models require data variables to be synchronized at every 16 ms as discused above. While there is a tendency of the MPD increasing as the system load increases because of the nature of microscopic simulation, all MPD values stay below 16 ms. From the results, we claim that the H2iLS is capable of real-time processing with an increase in the traffic volume until the road is full of vehicles.

**Figure 7:**
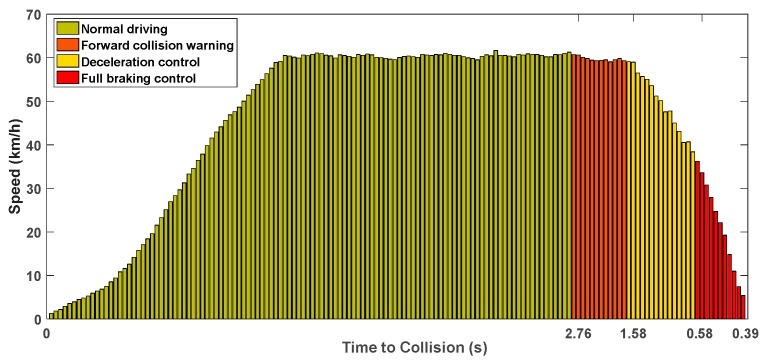
Speed change of the connected and non-automated vehicle (CNAV) as a function of time to collision (s) rounded to the two decimal places.

To evaluate the full braking control of the AEB service, a measure of the speed reduction is used. In the case of the full braking control, it indicates the difference between the speed after the vehicle completely stops (i.e., obviously zero) and the speed when the vehicle starts full braking control. The vehicle should experience the speed reduction greater than 15.8 km/h that is recommended by NHTSA [76]. We conduct this experiment eight times following the guidelines of Insurance Institute of Highway Safety (IIHS) [77]. Figure 7 shows one example of the results of eight trials to examine the driving speed change of the CNAV as a function of TTC.

It shows that the forward-collision warning, deceleration control, and full braking are triggered according to the change of the TTC value. During eight trials, the speed reduction in the full braking control ranges from maximum 36.238 km/h to minimum 32.2715 km/h and the mean speed reduction is 32.8646 km/h. They show that our AEB is well implemented to satisfy the speed reduction over 15.8 km/h as recommended by NHTSA.

As a result, the evaluation results clearly indicate that the H2iLS is able to perform the autonomous intersection management without suffering performance penalties while managing the road capacity and supporting at least the L2 of automation to provide the safety distance and the speeds.

## 5. Effectiveness of an Interactive ITCPS Framework

It is easy to point out that human behavior is one of the main causes of accidents, but it is difficult to prove that the cause of an accident is human error. It is impossible to remake the same situation in the real world and is too dangerous to re-enact it on the real road in order to obtain relevant data. Especially, in the event of an accident involving a CAV and a CNAV, it is even more difficult to demonstrate the technical insufficiencies of the CAV at an acceptable and reasonable level. There is also a lack of relevant data for the review of technical aspects necessary for legislation. Note that quantitative and qualitative data are essential to better understand human behavior in different situations. To obtain reliable results from the analysis, it requires gathering data from various participants in the same situation for each of the different scenarios. Hence, the interactive ITCPS framework should provide an environment to generate a reliable dataset of the human driver’s intentions in the complex system and be able to investigate the effects of human behavior on the transportation system.

In this regard, we examine the effectiveness of our framework as an experimental environment for the connected technology-based transportation research through a case study using the AIM application. First, the reliability of the variables is evaluated to determine if they can measure the same intentions [78]. Second, we investigate how the driving performance indicators representing the human behaviors vary depending on the V2V communication support during traveling, and then identify important indicators that may affect road safety by using a *t*-test. The third analysis is linear regression to estimate the dependency of the safety indicator on the identified driving performance indicators. The experiments in this section are conducted without the AEB function.

If a CNAV is not driven under the intersection coordination of the AIM, it needs to decide whether to maintain the current lane and velocity when approaching an intersection. To make the decision, a human driver should perform a series of centralized mental functions consisting of perception, intellection, emotion, and volition (PIEV) [79]. In transport engineering, one of the human factors associated with this PIEV process can be represented as perception-reaction time (PRT), which is defined as the minimum time required for a driver to react [80]. It is a significant parameter in road safety and design. Prior research shows that driver’s PRT is affected by the complexities involving drivers’ tendency, driving maneuvers, driving environments, and wireless communication capabilities [42,81,82]. Especially, since the V2X communication technology is intended to help drivers’ decision making such as acceleration choice and lane-changing maneuvers, it is expected that V2X communication could potentially improve drivers’ efficiency and response [43,81]. Hence, it is expected that the combination of the intersection coordination function and the V2X communication capability helps reduce or avoid crashes at an intersection by decreasing the driver’s PRT. However, under the mixed traffic environment, capturing the effects of these technologies on driving is challenging due to the uncertainty of human decisions. It is also difficult to quantify the uncertainty (e.g., diversity) of human behaviors and the effects on road safety depending on the various factors such as the complex interaction between CAVs and CNAVs, the intersection protocol, and the V2V communication. To the best of our knowledge, it is the first attempt to identify factors affecting the driver’s PRT and road safety in the mixed traffic environment with un-signalized intersections.

### 5.1. Measured Variables and Reliability Analaysis

The group of 12 subjects who participated in the tests consists of 75% males and 25% of females with an age range between 28 and 39 years; the limited number is deemed adequate given that the purpose here is to demonstrate the capabilities of the framework. The adequacy of this small number will be assessed by using the Kolmogorov and Smirnov normality test. They are involved in generating paired samples under two scenarios of the mixed traffic environment and repeatedly traveled 10 times under each scenario. These measurements are intended to reduce errors caused by the driver unacquainted with both H2iLS and experimental scenarios. Enhancing their competence in the virtual driving is expected to increase the reliability of the data. One scenario is to drive without the V2X communication and intersection protocol support. To minimize the drivers’ carelessness in this experiment, all subjects are strongly required to comply with the instruction presented on the HUD device while driving. The second scenario is to drive under V2V communication and intersection protocol support. During driving, every subject is informed of the intersection coordination model’s decision to cross the intersection immediately or to wait. In these scenarios, the driver’s PRT can be varied according to a level of drivers’ concentration such as keeping eyes on the road and the HUD device. A speed limit on this road of H2iLS is set to 60 km/h. The length of the road section to the un-signalized intersection is designed to be 300 m.

In our experiment, we construct a sampling dataset with records representing human driver information, driving performance indicators, and a driving condition. The fields about the human driver information consist of the gender and the age of the drivers. The fields of driving performance indicators are composed of transit time, the number of braking events, driver’s PRT, a safety indicator, and an average driving velocity. The driving condition field indicates if the V2V communication is supported during driving. If V2V communication is not supported, the intersection protocol does not operate in a vehicle. The transit time is defined as the time from the moment a driver starts to travel until the moment the driver completely crosses the intersection. A braking event (BE) is triggered whenever a driver puts on the brake pedal during the travel. The number of BEs is associated with the driver’s uncertainty. The PRT represents a total time taken for the PIEV process required for braking. More specifically, the PRT is measured by a time interval between the time point with the maximum change in the deceleration triggered after full acceleration and the time point at which the driver starts putting a brake pedal. In our tests, the mean PRT is computed using the sum of the PRTs measured for each BE during the driving. Since the safety indicator represents a crash ratio per driver during traveling, a low value of the safety indicator means high road safety. The average driving velocity is calculated using a series of data measured as the vehicle’s velocity during traveling.

The values measured under the two scenarios are summarized in Table 3. The simple comparison of the measurements in the two scenarios shows that the PRT of the drivers with connectivity is noticeably improved. In addition, the crash ratio and the number of BEs become lower than those of the drivers with no connectivity. Decreasing tendencies of these values are consistent, regardless of age and gender. It is expected that the connectivity decreases the transit times of the CNAVs. However, it is interesting to note that, except for data of the female participants, the transit time with the connectivity support has not improved significantly, compared to that without connectivity support. It seems to be due to the transit delays of the CNAVs with connectivity support, which is caused by the intersection protocol.

To investigate the adequacy of the specified variables in our case study, we employ reliability analysis with Cronbach’s alpha coefficient for measured data. The reliability indicates that there is no difference in measurements when the specified variables observing the same intention of the drivers are measured multiple times. A Cronbach alpha provides a measure of the internal consistency of a test and is an index of reliability [82,83]. In other words, Cronbach’s alpha coefficient has a high value when the variables consistently capture one particular characteristic. Its alpha coefficient of greater than or equal to the suggested value of 0.60 is interpreted as acceptable [84,85]. For analysis variables of the V2V communication support, PRT, and crash ratio, the Cronbach alpha coefficient is 0.717. For analysis variables of the transit time and BEs, its alpha coefficient of 0.604 provides an acceptable lower bound for the reliability coefficient. Therefore, there is the reliability among the variables. The variables designed in our experiments are valid because of the reliability among the variables [78].

### 5.2. Analysis of Driving Performance Affected by V2V Communication Support

In this subsection, we identify the driving performance indicators that are affected by V2V communication support by using a paired *t*-test. First, we set three independent variables with two categories. The independent variables are composed of the age of drivers (i.e., 20 s and 30 s), the gender of drivers (i.e., male and female), and if V2V communication is supported (i.e., no and yes). The V2V communication support is denoted as VCS. No-VCS denotes no support for V2V communication. In the case of no-VCS, both V2V communication and the intersection protocol do not operate in a vehicle. Second, we set dependent variables as the mean of the transit time denoted as μTT, the mean number of the BEs denoted as μBE, the mean of the PRTs denoted as μPRT, and a safety indicator denoted as μSI. We use the SPSS (PASW) version 18.0 tool to carry out a paired *t*-test that examines the mean difference between dependent variables under V2X communication support. In parametric statistics, the applicability of tests (e.g., *t*-test, analysis of variance, and regression) is based on the assumption that the sampling distribution is normal. This property is related to external validity indicating how well the analysis results are generalized in the real world. However, their applicability is limited by the size of the samples available for the analysis. Furthermore, since we have a small dataset with 12 samples, we need to verify the assumption of normality before using some parametric tests. In order for the paired *t*-test to be valid, the differences between paired samples should be approximately normally distributed.

We employ a Kolmogorov–Smirnov normality test that examines if the observations are normally distributed. A null hypothesis is that the distribution of the PRTs follows the normal distribution. The results of the normality test for our sampling dataset are summarized in Table 4.

Since the *z*-value of Kolmogorov–Smirnov is 0.157 and the returned *p*-value is 0.200, this test accepts the null hypothesis at the 5% significance level. We also perform this normality test for μTT, μBE, and μSI, respectively. The distributions of dependent observations are not different from normal distribution because all *p*-values returned for individual variables are greater than the default significance level (α=0.05).

In the paired *t*-test, a null hypothesis is that there is no pairwise difference of V2X communication support at the 5% significance level. We perform the paired *t*-test for the four observed μTT, μBE, μPRT, and μSI. Since the returned *t*-value and *p*-value of μPRT are 6.803 and 0.000029, respectively, the *t*-test rejects the null hypothesis and there is a statistically significant difference in the average PRTs between VCS and no-VCS. As the mean values of μPRT under no-VCS and VCS are 2.0115 ± 0.62909 and 0.8588 ± 0.28558, respectively, μPRT under VCS is reduced. The results of the dependent variables except for those of the transit time (μTT) indicate that the *t*-test also rejects the null hypothesis. The results of μTT indicate that there is no statistically significant difference between no-VCS and VCS. The results of the paired *t*-test are shown in Table 5. Consequently, there are statistically significant differences in the perception-reaction time, the number of braking times, and the safety indicator depending on the support of V2V communication.

### 5.3. Analysis of Factors Affecting Driving Safety Level

We identify factors that affect road safety and estimate the dependency indicating how much driving safety is affected by them. The ratio of crashes that a driver causes is defined as a criterion variable (μSI). The predictor variables are defined as all driving performance indicators (μPRT, μTT, and μBE), the human driver information (age and gender), and the driving condition (VCS and no-VCS). To convert to the continuous variables, dummy variables are re-defined for the gender and the driving condition variables corresponding to the categorical variable. The dummy variable for the driving condition is denoted as δVCS. The age of drivers is given with an age range between 28 to 39 years as a form of continuous variables.

We first perform the correlation analysis for linear regression to identify the relationship between each predictor variable and the criterion variable. If there is no relationship between a given predictor and the criterion variable, we exclude the predictor variable from the linear regression analysis. The results of the Pearson’s correlation analysis with coefficients and p-values show that only VCS, PRT, and BEs have a correlation with the criterion variable because the p-values of VCS, PRT, and BEs are $3.543\times 10^{-6}$, 0.029, and 0.002, respectively. Therefore, only the three selected variables are considered as predictor variables for the multiple-linear regression and a stepwise method is employed to find out the regression equation by with the predictor variables [86]. The regression analysis for determining the criterion variable $\hat{y}$ of the safety indicator (μSI) yields the following equation.

(19) $$\hat{y}=0.417-0.303X_{1}-0.092X_{2},$$

where $X_{1}$ and $X_{2}$ are the V2V communication support and the PRT, respectively. The factors that proved to be significant in relation to the safety indicator are the V2V communication support and the PRT, but the number of the BEs is consequently excluded from this analysis by the stepwise method. The regression equation shows that V2V communication capability tends to further decrease the crashes at the un-signalized intersection. It also shows that large PRTs may help ensure intersection safety. The shorter the driver’s PRT, the easier it is to avoid a sudden collision with a preceding vehicle or an obstacle. However, in our experiments, it seems that the drivers do not push the brake pedal immediately after recognizing the risk of the intersection ahead since passing safely through the intersection is not a critical risk. The drivers tend to pay close attention to crossing the un-signalized intersection by letting up the accelerator rather than immediately stepping on the brake. In this regard, the large PRT represents the high level of the driver’s prudence watching the situation. Therefore, the vehicle is likely to safely cross the un-signalized intersection when the driver has a large PRT.

Although it is shown that the BEs are related to the safety indicator by Pearson’s correlation analysis, the dependency between them cannot be estimated. This is because it is not statistically significant for the BEs to have an impact on the crash ratio from the result of the multiple linear regression. The regression coefficients related to Equation (19) are summarized in Table 6.

The significance of the coefficients presented in Table 6 is tested with a one-way ANOVA test that provides the information about the levels of the significance of the regression model [87]. The results of the ANOVA are shown in Table 7. In this analysis, a null hypothesis is that all predictor variables (μPRT, μTT, and μBE) affect a given criterion variable (μSI) and an alternative hypothesis is that at least one predictor variable has an effect on a given criterion variable. The *f*-value of 27.367 is larger than $F_{3,20;0.05}$ value (i.e., the *f*-value of 3.10) in the F-distribution Table and the *p*-value as shown in Table 7 is under the α=0.05 level of significance. Therefore, the null hypothesis is rejected and the regression equation of Equation (19) is found to be significant.

To ensure the reliability of our regression analysis, we present the information about multicollinearity and autocorrelation. The multicollinearity means the predictor variables are highly correlated. The autocorrelation occurs if the regression model is incorrectly specified. If two properties exist in the regression model, the estimated coefficient is likely not to be statistically significant. In the multicollinearity analysis, the variance inflation factor (VIF) for the predictor variables is 2.518. Since our value is lower than 10, there is no multicollinearity between variables. The autocorrelation analysis is achieved by the Durbin–Waston test. Our result of the Durbin–Waston test is 1.881. We examine the presence of the autocorrelation by comparing our Durbin–Waston value with the value in the table of Savin and White with the α=0.05 level of significance [88,89]. Our value is higher than the upper value of 1.54639 derived from the Savin and White table for 24 samples and three terms [88]. It indicates that the predictor variables are significant since our model does not have any autocorrelation. Therefore, our regression model is valid using the specified predictors. The variations in the predictor variables of (19) cover 69.6% of the variations in the criterion variable because the coefficient of determination $R^{2}$ for our regression model is obtained to be 0.696 as shown in Table 8.

### 5.4. Discussion

From a simple comparison of the measurements in two scenarios of VCS and no-VCS, it is clear that V2V communication and intersection protocol support tend to reduce the mean PRT, the mean crash ratio, and the mean number of brake events, regardless of the gender, age, and mean velocity. In the scenario of VCS, we expected the mean transit time at the un-signalized intersection to be less than that of no-VCS. However, the mean transit time is not significantly reduced since the V2V communication-based intersection protocol coordinates vehicles that will cross the intersection, causing most vehicles to slow down. Only in the case of the female participants, the mean transit time is significantly reduced by the V2V communication-based intersection protocol. It seems that they drive more carefully than male participants when driving without connectivity.

The results of the paired *t*-test show that V2V communication and intersection protocol support lead to a significant difference in the PRT. It also makes noticeable differences in the number of BEs and the crash ratio. Among driving performance indicators (PRT, BE, and transit time), the human driver information (age and gender), and the driving condition (VCS and no-VCS), the indicators affecting the road safety are PRT and VCS. We figured out two important facts from this analysis. First, high PRT has a positive effect on road safety irrespective of VCS. Especially, to pass through the un-signalized intersection, the drivers tend to watch how the situation develops after identifying the risk, which leads to an increase in PRT. In other words, PRT represents the level of driver’s prudence to take a deliberate action (i.e., stop a human-driven car by stepping on the brake). Second, road safety is improved by V2V communication and intersection protocol support that have the capability to reduce the mean PRT.

Consequently, from our case study, we verified the assumption that the combination of the intersection protocol and V2X communication capability helps reduce or avoid crashes at an intersection by decreasing the driver’s PRT. We also found out that, irrespective of V2V communication support, the road safety may be affected by the driver’s prudence observed as PRT. Furthermore, since the H2iLS demonstrates the consistency between data derived from multiple human drivers, it is expected to be useful with generating and exploiting a reliable dataset of human drivers’ intention in complex systems.

## 6. Conclusions

As automation in vehicles progresses rapidly, the associated human factors become more complex and it is difficult to predict driver’s behaviors on the road. For investigating the associated human factors, it is essential to provide an environment that can support the interaction between autonomous vehicles and human drivers under mixed traffic conditions. While there has been earlier work in these fields, such interactions have not been studied extensively, and it is not yet clear what should happen and what should change to minimize unforeseen risks and enhance road safety in the mixed traffic. In addition, since many studies for the ITCPS environment are conducted independently in their fields, there is a lack of an integrated, comprehensive environment for ITCPS. It is not easy to build such an environment since it requires significant efforts to merge various disciplines and technologies to integrate heterogeneous sub-systems. The dynamic nature of ITCPS, combined with coexistence and interactions with human drivers, is the major challenge in the design of an interactive ITCPS framework.

To address this challenge, we propose an interactive ITCPS framework to build the core of a virtual ITCPS environment. The component-based design of the interactive ITCPS framework can provide the standardization, efficiency, scalability, and reusability of this environment. In our framework, all vehicles are designed to be connected through the standardized V2V communication for interaction. In addition, to investigate the interaction representing the effects of human factors, a form of human and hardware-in-the-loop system is imperative. The driver’s reaction (behavior) is provided as the necessary feedback to close the loop with the coordinated environment in a timely manner. In the H2iLS, a human driver can travel using CNAVs equipped with the steering wheel, brake pedal, gears, and acceleration pedal as well as in-vehicle networks for vehicle control, while all CAVs are generated as a form of a virtual and visual object. We have conducted performance evaluation to assess how well the implemented system behaves according to the interactive ITCPS framework design. The results of the performance evaluation demonstrate that our framework supports real-time connectivity and performs the behavior models by component interfaces. In addition, a case study has shown that the H2iLS is useful as an experimental ITCPS environment that has the capability to produce reliable data by showing the internal consistency between data measured from human drivers. The results of the case study validate our assumption that autonomous intersection coordination and V2V communication help reduce and avoid collisions at the un-signalized intersection by reducing the drivers’ PRTs.

The proposed framework provides a flexible way to evaluate the effectiveness of the various services and technologies for the realization of the ITCPS by eliminating potential risks on real roads. It can be used as a developing tool for improving the technologies for the next generation of ITS. It is available as a platform to efficiently analyze the relationship between the various factors such as traffic situations, road environments, driving conditions, and driving styles. We expect that the proposed framework will be utilized for human factors research, and with necessary extensions, it may enable the drivers to experience the diversity of vehicle automation and how the automation functionality should interact with humans in physical vehicles. Furthermore, it enables human factors analysis to be conducted in a safe way. We plan to expand the framework a little bit further in the communication model to investigate the effect of communication performance (e.g., packet loss, transmission delay, and end-to-end delay) on interactions between vehicles and to study various security issues that may occur in vehicular communication. To handle those metrics, we will add some features of a data link layer. After that, we will distribute the packaged components of our framework to provide an environment to evaluate the functionality of V2V-based safety services for cooperative intelligent transportation systems with vehicle automation. We plan to distribute the packaged components of our framework to provide an environment to evaluate the functionality of V2V-based safety services for cooperative intelligent transportation systems with vehicle automation.

## Figures and Tables

**Table 1:** Summarized data elements for each data frame.

Data Frame	Element	Description
Management	id	Vehicle ID
	secMark (s)	Message generation time (s)
	secMark (ms)	Message generation time (ms)
	msgCnt	Number of messages sent
Position	Latitude	Current latitude of the vehicle
	longitude	Current longitude of the vehicle
	elevation	Current elevation of the vehicle
	positonAccuracy	Location accuracy
Vehicle Size	length	Vehicle length
	width	Vehicle width
	height	Vehicle height
Steering Wheel	heading	Heading of the vehicle
	angle	Steering wheel angle
Movement	Speed	Speed
	latAccel	Longitudinal acceleration
	longAccel	Lateral acceleration
	vertAccel	Vertical acceleration
Vehicle Status	transmissionStatus	Type of the node role
	brakeStatus	Brake system status
Safety Extension	length	Length of extension data
	type	Application type
	msgID	Message identifier for application-specific data
	data	Application-specific data

**Table 2:** Mean transmission time interval and mean processing delay in milliseconds measured in the H2iLS (human and hardware-in-the-loop system).

Traffic Volume	Transmission Time Interval		Processing Delay	
	Mean Value (ms)	Standard Deviation (ms)	Mean Value (ms)	Standard Deviation (ms)
50	100.0226	0.003227	3.736723	0.082205
100	100.0771	0.000652	3.786558	0.089956
150	100.0937	0.001789	3.78358	0.116582
200	100.1299	0.001476	3.913733	0.10183
250	100.1784	0.030182	4.062175	0.091888
300	100.2017	0.034753	4.180116	0.114979
350	100.2028	0.038043	4.339588	0.117219
400	100.2261	0.03531	4.56229	0.091812
450	100.2421	0.066369	4.790223	0.121612
500	100.2763	0.081003	5.140928	0.122745
540	100.3872	0.22573	5.798167	0.151041

**Table 3:** Summary of variables measured in each scenario.

Variable		No V2V Communication Support (Scenario A)				V2V Communication Support (Scenario B)			
		Mean Transit Time	Mean PRT ^1^	Mean Braking Events ^1^	Mean Crash Ratio ^1^	Mean Transit Time	Mean PRT ^1^	Mean Braking Events ^1^	Mean Crash Ratio ^1^
Age ^2^	20 s	3.682	2.275	2.528	0.194	3.583	0.981	1.505	0.037
	30 s	3.647	1.700	3.110	0.271	3.427	0.814	1.538	0.033
Gender	Male	3.015	2.025	2.927	0.236	3.581	0.838	1.625	0.025
	Female	4.066	1.970	2.494	0.222	2.216	0.920	1.208	0.067
Mean Velocity (km/h)	<30 ^3^	4.855	1.933	4.000	0.275	3.941	0.998	1.483	0.053
	30–40 ^4^	2.921	2.189	1.980	0.185	2.874	0.581	1.597	0
	40–50 ^5^	3.315	1.821	2.643	0.255	-	-	-	-

^1^ Rounded to the three decimal places; ^2^ Six subjects in the 20s age and six subjects in the 30s age; ^3^ Four subjects in Scenario A and eight subjects in Scenario B; ^4^ Five subjects in Scenario A and four subjects in Scenario B; ^5^ Three subjects in Scenario A and 0 subjects in Scenario B.

**Table 4:** Results of Kolmogorov–Smirnov normality test.

Variable	*p*-Value ^1^
μPRT	0.2
μTT	0.153
μBE	0.2
μSI	0.2

^1^ The default significance level is 0.05 and a degree of freedom is 12.

**Table 5:** Results of the paired *t*-test.

Variable	*t*-Value ^1^	*p*-Value
μPRT	6.803	0.000029
μTT	0.321	0.754
μBE	3.798	0.003
μSI	5.750	0.00012

^1^t=1.796 when $t_{0.95}$ and df=11 in the *t*-value table.

**Table 6:** Coefficients of a criterion variable, μSI.

Model	Beta	Std. Error	*t*-Value	*p*-Value
Constant	0.417	0.066	6.333	2.797 × 10^−6^ ^1^
$X_{1}$	−0.303	0.046	−6.565	1.675 × 10^−6^ ^1^
$X_{2}$	−0.092	0.031	−2.949	0.008

^1^ Rounded to the three decimal places.

**Table 7:** ANOVA of a criterion variable, μSI.

Model	Sum of Squares	Degree of Freedom	Mean Square	*f*-Value	*p*-Value
Regression	0.278	2	0.139	27.367	1.415 × 10^−6^ ^1^
Residual	0.107	21	0.005		
Total	0.384	23			

^1^ Predictor variables: δVCS and μPRT, and rounded to the three decimal places.

**Table 8:** Model summary of a criterion variable, μSI.

*R*	$R^{2}$	Adjusted $R^{2}$
0.850 ^1^	0.723	0.696

^1^ Predictor variables: δVCS and μPRT.

## References

[1] Lee E. (2015). The past, present and future of cyber-physical systems: A focus on models. Sensors.

[2] Belezina J. Autonomo-Fully Autonomous Vehicle Designed for the Year 2030. https://newatlas.com/autonomo-fully-autonomous-vehicle-designed-for-the-year-2030/20529.

[3] Delhi S.I.N. (2016). Automotive revolution & perspective towards 2030. Auto Tech Rev..

[4] Wharton, Long Road Ahead: The Promise and Perils of Self-Driving Cars. https://knowledge.wharton.upenn.edu/article/self-driving-cars/.

[5] Lowry T. Americans Fearful of Self-Driving Cars: New AAA Survey. https://skift.com/2018/05/22/americans-fearful-of-self-driving-cars-new-aaa-survey/.

[6] Kramer S. Most Americans Are Uncomfortable with Self-Driving Cars. https://www.schmidtkramer.com/blog/americans-mistrust-self-driving-cars.html.

[7] Road Safety GB (RSGB), More than Half of UK Drivers ‘Uncomfortable’ with Self-Driving Cars. http://roadsafetygb.org.uk/news/n-a-5355/.

[8] McGougan J. Uphill Road ahead for Autonomous Vehicles. http://www.iicom.org/images/iic/intermedia/Jan-2017/im-jan2017-uphill-road-ahead.pdf.

[9] Mlot S. Distracted Driver Rear-Ends Google Self-Driving Car. https://www.pcmag.com/news/336101/distracted-driver-rear-ends-google-self-driving-car.

[10] Fingas J. Google Self-driving Car Crashes into a Bus (Update: Statement). https://www.engadget.com/2016/02/29/google-self-driving-car-accident/.

[11] Islam M.B., Kanitpong K. (2008). Identification of factors in road accidents through in-depth accident analysis. IATSS Res..

[12] Abbink D.A., Mulder M., Boer E.R. (2012). Haptic shared control: Smoothly shifting control authority?. Cognit. Technol. Work.

[13] Kaber D.B., Liang Y., Zhang Y., Rogers M.L., Gangakhedkar S. (2012). Driver performance effects of simultaneous visual and cognitive distraction and adaptation behavior. Transp. Res. Part F Traffic Psychol. Behav..

[14] Lu S.A., Wickens C.D., Prinet J.C., Hutchins S.D., Sarter N., Sebok A. (2013). Supporting interruption management and multimodal interface design: Three meta-analyses of task performance as a function of interrupting task modality. Hum. Factors.

[15] Beller J., Heesen M., Vollrath M. (2013). Improving the driver–automation interaction: An approach using automation uncertainty. Hum. Factors.

[16] Larsson A.F. (2012). Driver usage and understanding of adaptive cruise control. Appl. Erg..

[17] Carlson M.S., Jill L., Drury M.D., Hyangshim K., Holly A.Y. Identifying factors that influence trust in automated cars and medical diagnosis systems. Proceedings of the 2014 AAAI Spring Symposium Series.

[18] Blair K., Sandry J., Rice S. (2012). An expansion of system wide trust theory using in-vehicle automation. Proceedings of the Human Factors and Ergonomics Society Annual Meeting.

[19] Merat N., Jamson A.H., Lai F.C., Carsten O. (2012). Highly automated driving, secondary task performance, and driver state. Hum. Factors.

[20] Neubauer C., Matthews G., Langheim L., Saxby D. (2012). Fatigue and voluntary utilization of automation in simulated driving. Hum. Factors.

[21] Son S. (2017). Effect of Human Reactions at Signalized Intersections on Intersection Efficiency and Safety. Advances in Human Aspects of Transportation.

[22] Champion A., Mandiau R., Kolski C., Heidet A., Kemeny A. Traffic generation with the SCANeR II simulator: Towards a multi-agent architecture. Proceedings of the Driving Simulation Conference.

[23] Zhao Y., Sadek A.W. (2013). Computationally-efficient approaches to integrating the MOVES emissions model with traffic simulators. Proced. Comput. Sci..

[24] Hou Y., Zhao Y., Wagh A., Zhang L., Qiao C., Hulme K.F., Liu X. (2015). Simulation-based testing and evaluation tools for transportation cyber–physical systems. IEEE Trans. Veh. Technol..

[25] Punzo V., Ciuffo B. (2010). Integration of driving and traffic simulation: Issues and first solutions. IEEE Trans. Intell. Transp. Syst..

[26] That T.N., Casas J. (2011). An integrated framework combining a traffic simulator and a driving simulator. Proced. Soc. Behav. Sci..

[27] Jeihani M., NarooieNezhad S., Kelarestaghi K.B. (2017). Integration of a driving simulator and a traffic simulator case study: Exploring drivers’ behavior in response to variable message signs. IATSS Res..

[28] Parliament E. Directive 2010/40/EU of the European Parliament and of the Council of 7 July 2010 on the Framework for the Deployment of Intelligent Transport Systems in the Field of Road Transport and for Interfaces with Other Modes of Transport. http://www.cita.lu/uploads/its/Directive_2010-40-EU_EN.pdf.

[29] (2010). Road Traffic Management Strategy.

[30] ReportBuyer The Automotive Navigation Systems Market, in Terms of Value, Generated $20,953 Million in the Year 2016. https://www.prnewswire.com/news-releases/the-automotive-navigation-systems-market-in-terms-of-value-generated-20953-million-in-the-year-2016–300580365.html.

[31] Debernard S., Chauvin C., Pokam R., Langlois S. (2016). Designing human-machine interface for autonomous vehicles. IFAC Pap. Online.

[32] Sentouh C., Popieul J.C., Debernard S., Boverie S. (2014). Human-machine interaction in automated vehicle: The abv project. IFAC Proc. Vol..

[33] Favarò F.M., Nader N., Eurich S.O., Tripp M., Varadaraju N. (2017). Examining accident reports involving autonomous vehicles in California. PLoS ONE.

[34] Malin F., Norros I., Innamaa S. (2019). Accident risk of road and weather conditions on different road types. Accid. Anal. Prev..

[35] Persaud B.N., Retting R.A., Garder P.E., Lord D. Observational before after Study of the Safety Effect of US. Roundabout Conversions Using the Empirical Bayes Method. https://pdfs.semanticscholar.org/a3ec/b99fdbb502a96472d723fad9a04ec12d4dd2.pdf.

[36] De Brabander B., Vereeck L. (2007). Safety effects of roundabouts in Flanders: Signal type, speed limits and vulnerable road users. Accid. Anal. Prev..

[37] Čolić A., Marques O., Furht B. (2014). Driver Drowsiness Detection: Systems and Solutions.

[38] McCartt A.T., Ribner S.A., Pack A.I., Hammer M.C. (1996). The scope and nature of the drowsy driving problem in New York State. Accid. Anal. Prev..

[39] Rosca J., Ugalde I., Songchitruksa P., Sunkari S. (2017). CONVAS: Connected Vehicle Assessment System for Realistic Co-simulation of Traffic and Communications. Netw. Simul. Intell. Transp. Syst..

[40] Bogdan P., Marculescu R. A fractional calculus approach to modeling fractal dynamic games. Proceedings of the 2011 50th IEEE Conference on Decision and Control and European Control Conference.

[41] Siemens Traffic Prediction–Aimsun. https://new.siemens.com/global/en/products/mobility/road-solutions/traffic-management/strategic-management-and-coordination/traffic-prediction.html.

[42] Talebpour A., Mahmassani H.S. Modeling acceleration behavior in a connected environment. Proceedings of the Midyear Meetings and Symposium Celebrating 50 Years of Traffic Flow Theory.

[43] Talebpour A., Mahmassani H.S. Influence of Autonomous and Connected Vehicles on Stability of Traffic Flow. Proceedings of the Transportation Research Board 94th Annual Meeting.

[44] Vokrinek J., Schaefer M., Pinotti D. Multi-agent traffic simulation for human-in-the-loop cooperative drive systems testing. Proceedings of the 2014 International Conference on Autonomous Agents and Multi-Agent Systems.

[45] Vokřínek J., Janovský P., Faigl J., Benda P., Tango F., Pinotti D. A cooperative driver model for traffic simulations. Proceedings of the 2013 11th IEEE International Conference on Industrial Informatics (INDIN).

[46] Jin M., Lam S.H. A virtual-reality based integrated driving-traffic simulation system to study the impacts of intelligent transportation system (ITS). Proceedings of the 2003 International Conference on Cyberworlds.

[47] Schmidt R., Assmann U., Biegler P., Kramer R., Lockemann P.C., Rolker C. The interrelatedness of component-oriented systems and workflow-management. Proceedings of the Workshop on Composition Software Architect.

[48] Brazier F.M., Jonker C.M., Treur J. (2002). Principles of component-based design of intelligent agents. Data Knowl. Eng..

[49] Chaudron M., Larsson S., Crnkovic I. (2005). Component-based development process and component lifecycle. J. Comput. Inf. Technol..

[50] He J., Li X., Liu Z. (2005). Component-based software engineering. International Colloquium on Theoretical Aspects of Computing.

[51] Bass L., Clements P., Kazman R. (2003). Software Architecture in Practice.

[52] DSRC Committee (2015). Dedicated Short Range Communications (DSRC) Message Set Dictionary.

[53] Bennett J. (2010). Open Street Map.

[54] CityEngine E. 3D Modeling Software for Urban Environments. https://proj.org/about.html.

[55] Urbanek S. (2008). proj4: A Simple Interface to the PROJ. 4 Cartographic Projections Library (R package version 1.0-4).

[56] Messer C.J., Fambro D.B. Critical lane analysis for intersection design. Proceedings of the 56th Annual Meeting of the Transportation Research Board.

[57] Berry D.S., Gandhi P.K. (1973). Headway approach to intersection capacity. Highw. Res. Rec..

[58] Mathew T. (2014). Design priciples of traffic signal. Transp. Syst. Eng..

[59] Chang G.L., Joseph J. Empirical Investigation of Intersection Traffic Patterns Under Maryland Driving Populations. https://www.roads.maryland.gov/OPR_Research/MD-01-Analysis-of-the-Critical-Lane-Volume-Method-at-Signalized-Intersections.pdf.

[60] Hoogendoorn S., Knoop V. (2013). Traffic flow theory and modelling. Transp. Syst. Transp. Policy.

[61] Newell G.F. (2002). A simplified car-following theory: A lower order model. Transp. Res. Part B.

[62] Ro J.W., Roop P.S., Malik A., Ranjitkar P. (2017). A formal approach for modeling and simulation of human car-following behavior. IEEE Trans. Intell. Transp. Syst..

[63] Stolfi D.H., Alba E. (2018). Generating realistic urban traffic flows with evolutionary techniques. Eng. Appl. Artif. Intell..

[64] Azimi R., Bhatia G., Rajkumar R., Mudalige P. (2013). V2v-intersection management at roundabouts. SAE Int. J. Passeng. Cars Mech. Syst..

[65] Chen S.E., Williams L. View interpolation for image synthesis. Proceedings of the 20th Annual Conference on Computer Graphics and Interactive Techniques.

[66] Manning R.A., Dyer C.R. Interpolating view and scene motion by dynamic view morphing. Proceedings of the 1999 IEEE Computer Society Conference on Computer Vision and Pattern Recognition (Cat. No PR00149).

[67] Nakagami M. The m-distribution—A General Formula of Intensity Distribution of Rapid Fading. Proceedings of the a Symposium Held at the University of California.

[68] Sharma S., Mishra R. (2015). A Simulation Model for Nakagmi-m Fading Channel with m. Power.

[69] Ziegler C. Automatic Emergency Braking Will Be Standard in Most Us Cars by 2022. https://www.theverge.com/2016/3/17/11253656/nhtsa-iihs-automatic-emergency-braking-agreement-2022.

[70] Euro NCAP Euro NCAP 2025 Roadmap: In Pursuit of Vision Zero. https://cdn.euroncap.com/media/30700/euroncap-roadmap-2025-v4.pdf.

[71] Hydén C. (1996). Traffic conflicts technique: State-of-the-art. Traffic Saf. Work Video Process..

[72] Minderhoud M.M., Bovy P.H. (2001). Extended time-to-collision measures for road traffic safety assessment. Accid. Anal. Prev..

[73] Bloecher H.L., Dickmann J., Andres M. Automotive active safety and comfort functions using radar. Proceedings of the 2009 IEEE International Conference on Ultra-Wideband, IEEE.

[74] Mangharam R., Weller D., Rajkumar R., Mudalige P., Bai F. Groovenet: A hybrid simulator for vehicle-to-vehicle networks. Proceedings of the 2006 Third Annual International Conference on Mobile and Ubiquitous Systems: Networking and Services.

[75] Math R., Mahr A., Moniri M.M., Müller C. OpenDS: A new open-source driving simulator for research. Proceedings of the International Conference on Automotive User Interfaces and Interactive Vehicular Applications.

[76] Forkenbrock G.J., Snyder A.S. NHTSA’s 2014 Automatic Emergency Braking Test. Track Evaluations. https://www.nhtsa.gov/sites/nhtsa.dot.gov/files/812166-2014automaticemergencybrakingtesttrackeval.pdf.

[77] IIHS (2013). Autonomous Emergency Braking Test Protocol (Version I).

[78] Tavakol M., Dennick R. (2011). Making sense of Cronbach’s alpha. Int. J. Med Educ..

[79] Budiati A. (2014). Reading Speed to Be Used For Highway Signs and Traffic Marking In Indonesia. Acad. Res. Int..

[80] Kuang Y., Qu X., Weng J., Etemad-Shahidi A. (2015). How does the driver’s perception reaction time affect the performances of crash surrogate measures?. PLoS ONE.

[81] Smith B.L., Park H., Hayat M.T. (2016). Connected Vehicle Enabled Freeway Merge Management–Field Test.

[82] Cronbach L.J. (1951). Coefficient alpha and the internal structure of tests. Psychometrika.

[83] Santos J.R.A. (1999). Cronbach’s alpha: A tool for assessing the reliability of scales. J. Ext..

[84] Norman G.R., Streiner D.L. (1994). Biostatistics: The Bare Essentials.

[85] Hume C., Ball K., Salmon J. (2006). Development and reliability of a self-report questionnaire to examine children’s perceptions of the physical activity environment at home and in the neighbourhood. Int. J. Behav. Nutr. Phys. Act..

[86] Ghani I.M.M., Ahmad S. (2010). Stepwise multiple regression method to forecast fish landing. Proced. Soc. Behav. Sci..

[87] SAS Institute Inc. Interpreting Results in Explanatory Modeling. https://www.jmp.com/en_us/statistics-knowledge-portal/what-is-multiple-regression/interpreting-results-in-explanatory-modeling.html.

[88] Savin N.E., White K.J. (1997). The Durbin-Watson test for serial correlation with extreme sample sizes or many regressors. Econom. J. Econom. Soc..

[89] Durbin J., Watson G.S. (1951). Testing for serial correlation in least squares regression. II. Biometrika.

